# The *Cryptococcus neoformans* titanide is a pathogenic cell type that arises from typical yeast cells in response to host conditions

**DOI:** 10.1371/journal.pbio.3004031

**Published:** 2026-10-12

**Authors:** Abigail M. Potton, Iana Kalinina, Gordon D. Brown, Maria Makarova, Sandra Catania, Dora E. Corzo-Leon, Elizabeth R. Ballou

**Affiliations:** 1 MRC Centre for Medical Mycology, University of Exeter, Exeter, United Kingdom; 2 Department of Metabolism and System Science, School of Medical Sciences, College of Medicine and Health, University of Birmingham, Birmingham, United Kingdom; University of Toronto, CANADA

## Abstract

Fungal morphogenesis is a major driver of disease outcome. For the opportunistic fungal pathogen *Cryptococcus neoformans*, extreme morphological heterogeneity within the lung environment drives dissemination, immune evasion, and drug resistance. One such cell type is the small, oval, titanide (2–4 µm). This poorly understood cell type is prevalent in lung histology as well as host-relevant in vitro conditions. In conditions that generate cellular heterogeneity, titanides comprise 4%–10% of cells 24 hours post-induction and by 72 hours are the dominant cell type. Despite their prevalence and the significance of cryptococcal morphogenesis for virulence, key questions pertaining to their origin, fate, and underlying biology remain unstudied. Using TEM, SEM, and fluorescence microscopy we demonstrate that titanides display distinct morphological features; they possess thin cell walls, single birth scars, and capsules, with reduced Pathogen-Associated Molecular Pattern exposure and altered distribution. These features distinguish titanides from previously described *C. neoformans* ‘small cells’ such as in vivo seed cells (6 µm, high cell-wall mannan content) and micro cells (round, <1 μm, with thick cell walls) among others. Using microfluidics, we reveal that titanides arise from yeast-like ‘typical’ mothers, highlighting the morphological plasticity of typical cells in host-relevant conditions. Upon birth, titanides have compact nuclei and do not immediately proliferate, but transfer to fresh media results in rapid swelling and cell division. Consistent with this, titanides are more resistant to some cell wall/membrane stressors and are an effective infectious propagule in a murine model. Together, these findings establish a definition and essential characterisation of the titanide cell type and provide new insight into a key player in *C. neoformans* cell biology and pathogenesis.

## Abstract 2

*Cryptococcus neoformans* cells can adopt many different phenotypes during infection. This study uses microfluidics to show that one such form, titanides, arise from typical yeast cells and form a distinct, stress-resistant infectious cell type important for adaptation and disease.

## Introduction

*Cryptococcus neoformans* is a basidiomycetous opportunistic fungal pathogen responsible for life-threatening pulmonary infections and subsequent meningitis, predominantly in immunocompromised individuals [1–3]. An emerging feature of *Cryptococcus* cell biology is its morphological plasticity: Although *C. neoformans* is predominantly studied as an encapsulated haploid budding yeast (5–7 µm), it has the capacity to induce dramatic changes in size and ploidy in response to environmental conditions [2,4,5].

In addition to morphological plasticity, cellular heterogeneity is a hallmark of cryptococcal disease [2,6,7]. During infection, *C. neoformans* can generate a range of yeast-like morphologies with substantial differences in total cell body and capsule size/structure that directly influence pathogenesis [2,4,5,7–11]. Most dramatically, upon exposure to the pulmonary environment a small proportion of cells can undergo an unusual morphological transition into large (>10 µm) uninucleate polyploids known as titan cells [2,4,5,12–15]. Titan cells comprise 5%–20% of the population depending on fungal isolate and physiological and in vitro culture conditions [2,5,8,9,16–19]. Additional in vivo cell types include ‘typical’ cells (5–7 µm), seed cells (6 µm, high cell wall mannan), viable but non-culturable (VBNC) cells (5 μm, viability dye positive, low culturability), and micro cells (round, <1 μm) [7,15,20,21]. The latter were collectively called ‘small cells’ in the literature to distinguish them from in vivo ‘typical cells’ (5–7 µm) and large titan cells (>10 µm) [6,7,10,22].

We previously identified a small cell morphology during in vitro culture and in the lungs of infected mice, which we termed titanides [2,18]. Titanides display distinct morphological properties: they are oval, small in cell size (2–4 μm) and possess thin cell walls [2]. These are morphologically distinct from seed and VBNC cells (which are larger and thick-walled), and from micro cells (which are smaller and thick-walled) [7,10]. Strikingly, cells consistent with titanides are apparent in human lung histological samples where titan and typical cells are present [11,23–25]. However, little is known about titanides, including how they arise, their role in stress resistance, and their role in disease progression.

Here, we carefully define titanides based on size, shape, cell wall, and capsule staining patterns and demonstrate that they are morphologically and biologically distinct from typical 5–7 μm cells and other ‘small cells’. Using microfluidics and live cell imaging, we show that titanides arise from typical-sized mothers, and not from titan cells. Additionally, we demonstrate that titanides have unique proliferation properties and a capacity for morphological plasticity that allows them to form yeast and titan cells in response to relevant culture condition. We demonstrate that, despite their thin cell walls, titanides display no increase in sensitivity to cell wall/membrane stressors compared to yeast and, in some contexts, display increased resistance. Importantly, we demonstrate that titanides are an efficient infectious propagule in a murine model, with equivalent pathogenicity to infection with yeast. Taken together, we suggest titanides are a distinct *C. neoformans* cell type and driver of pathogenesis that can promote fungal survival during infection.

## Results

### A morphologically distinct small cell type arises in host-relevant conditions

In vitro induced titan cultures generate heterogenous populations over time [2,9]. To better understand population structure changes over time, we assessed 72-hour induced cultures for key measures including size, shape, and total chitin content using automated image analysis (Fig 1). It was previously demonstrated in cultures recovered from mouse lung that, compared to typical cells, titan cells have significantly increased total chitin, revealed by calcofluor white (CFW) staining [15,26]. We confirmed this for in vitro-induced cells in 72-hour cultures, observing large- and intermediate-sized round cells with CFW fluorescence intensity consistent with titan (circularity ~1, high CFW) and typical (circularity ~1, medium-high CFW) cells (Fig 1A). However, there was also a third more elongated population exhibiting reduced fluorescence (circularity <1, low CFW) (Fig 1A). Analysis of circularity score for cells greater than or less than 4 µm revealed a significant difference in shape below the 4 µm cutoff (Fig 1B), and when CFW staining intensity across the three size populations was analysed to account for differences in cell size (Integrated Density), intensity of small oval cells was significantly reduced, while titan cells were significantly increased, compared to typical cells (Fig 1C and 1D). Examining cultures over time (5, 6, and 7 hours post-induction), the first small oval cell was observed between 6 and 7 hours using two different laboratory isolates (H99O, KN99) (S1A Fig). Analysis of H99 cultures at 24, 48, and 72 hours shows that small oval cells only account for 3.98% of the population by 24 hours post-induction and their numbers increase at 48 hours, but at 72 hours they are the dominant cell type (54.36%) (Fig 1E and 1F). A similar trend was observed for KN99 (S1B).

**Fig 1:**
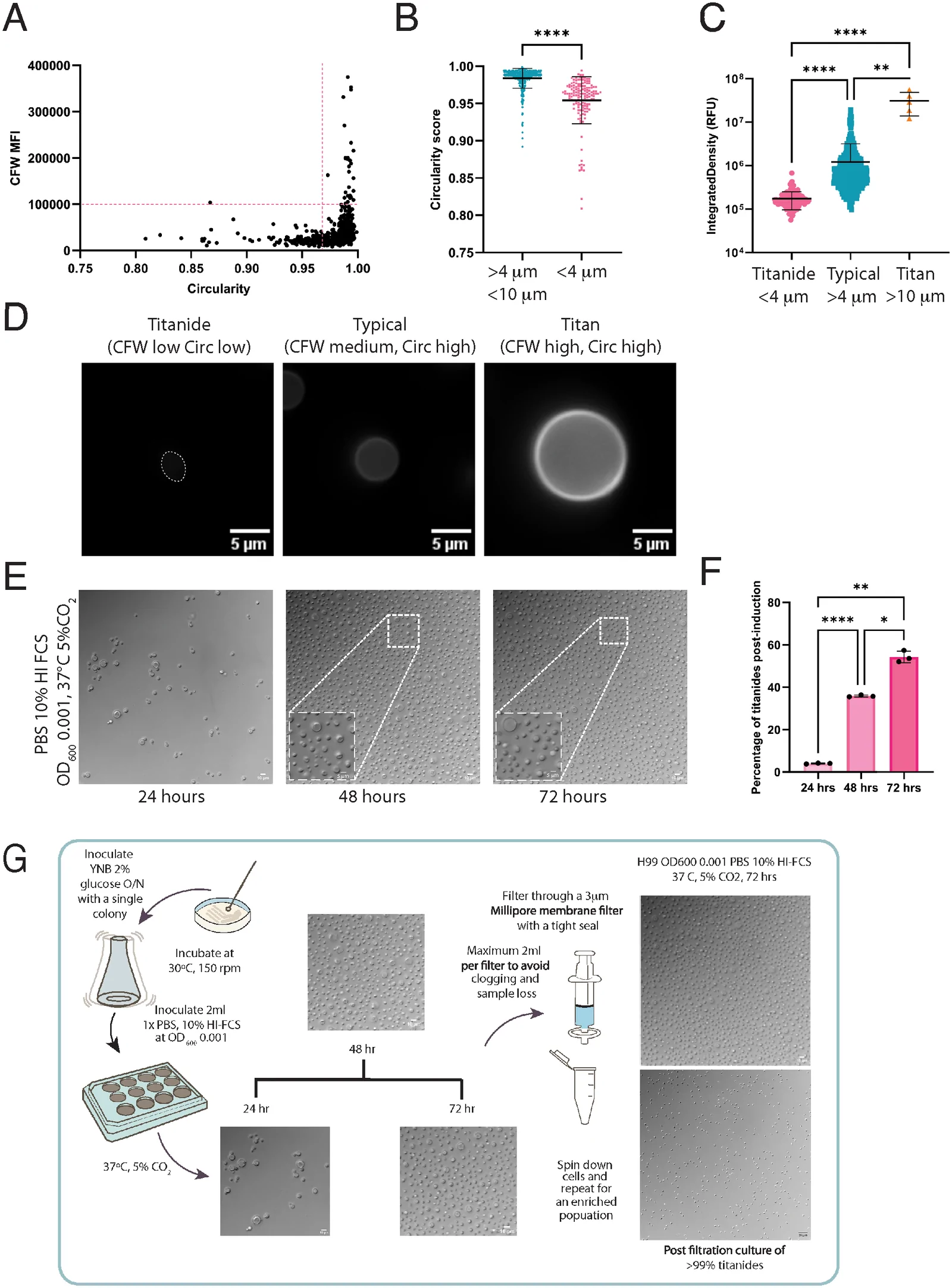
A morphologically distinct small cell arises from the heterogenous titan population. (**A**) 72-hour titanised cells were analysed for circularity vs. CFW Integrated Density, with dotted lines indicating distinct populations. **(B)** Cells from (A) were categorised by size and analysed for differences in circularity score. **(C)** Cells from (A) were categorised by size and analysed for differences in CFW Integrated Density (RFU, Relative Fluorescence Unit). **(D)** Representative cells from **(A, C)**. Scale bar 5 µm. **(E)** Representative images of in vitro titan cultures over 24, 48, and 72 hours. Scale bar 10 µm across first 3 images, 5 µm for zoom. **(F)** Percentage of titanides over time. **(G)** Schematic for filtering technique to isolate titanides, including representative images of 72-hour induced populations pre- and post-filtering. For all graphs, all cells in a given image were measured, with 3 images per sample, in biological triplicate, and mean and standard deviation indicated. ‘****’, *p* = < 0.0001, ‘**’, *p* = 0.0011, ‘*’, *p* = 0.0140, assessed by repeated measures ANOVA with Tukey’s multiple comparisons test. The data underlying this figure can be found at https://doi.org/10.6084/m9.figshare.31171789.

To specifically study these small oval cells, we developed a filtering technique to isolate pure cultures of cells <3 µm (Fig 1G), and confirmed by inspection of the well, demonstrating a >99% pure population (S2 Fig).

### Titanides are oval, 2–4 µm cells with thin cell walls, distinct from yeast and seed cells

Titanides were originally defined using TEM micrographs of in vitro titan cultures, a heterogenous mix of typical cells, titan cells, and a subpopulation of 2–4 µm oval cells [2]. We observed typical cells with walls on average 167 nm, while titan cell walls were on average 314 nm [2]. In contrast, cell walls of the 2–4 µm population were significantly thinner, at 56 nm [2]. To distinguish them from other *C. neoformans* ‘small cells’, we termed them “Titanides” after the Greek goddess siblings of the Titans, because they appear under host-relevant conditions that also induce titan cell formation [2]. To confirm that the small, oval, low CFW cells (Fig 1) are the titanide cell type, we performed TEM of pure cultures of small oval cells and determined the thickness of the cell wall compared to YPD-grown rich yeast cells. We additionally compared them to in vitro-induced “seed cells,” an in vivo*-*relevant “small cell” phenotype that has been hypothesised to be analogous to titanides [6,7]. As can be seen in Fig 2A, small oval cells have significantly thinner cell walls compared to YPD-grown cells (45.5 ± 10.3 nm versus 144.9 ± 25.5 nm, *p* = <0.0001) (Fig 2A and 2B). In contrast, seed cell wall thickness was increased beyond YPD-grown cells 212.4 ± 38.1 nm, consistent with reports that these cells have high mannan content compared to typical cells (Fig 2A and 2B) [7].

**Fig 2:**
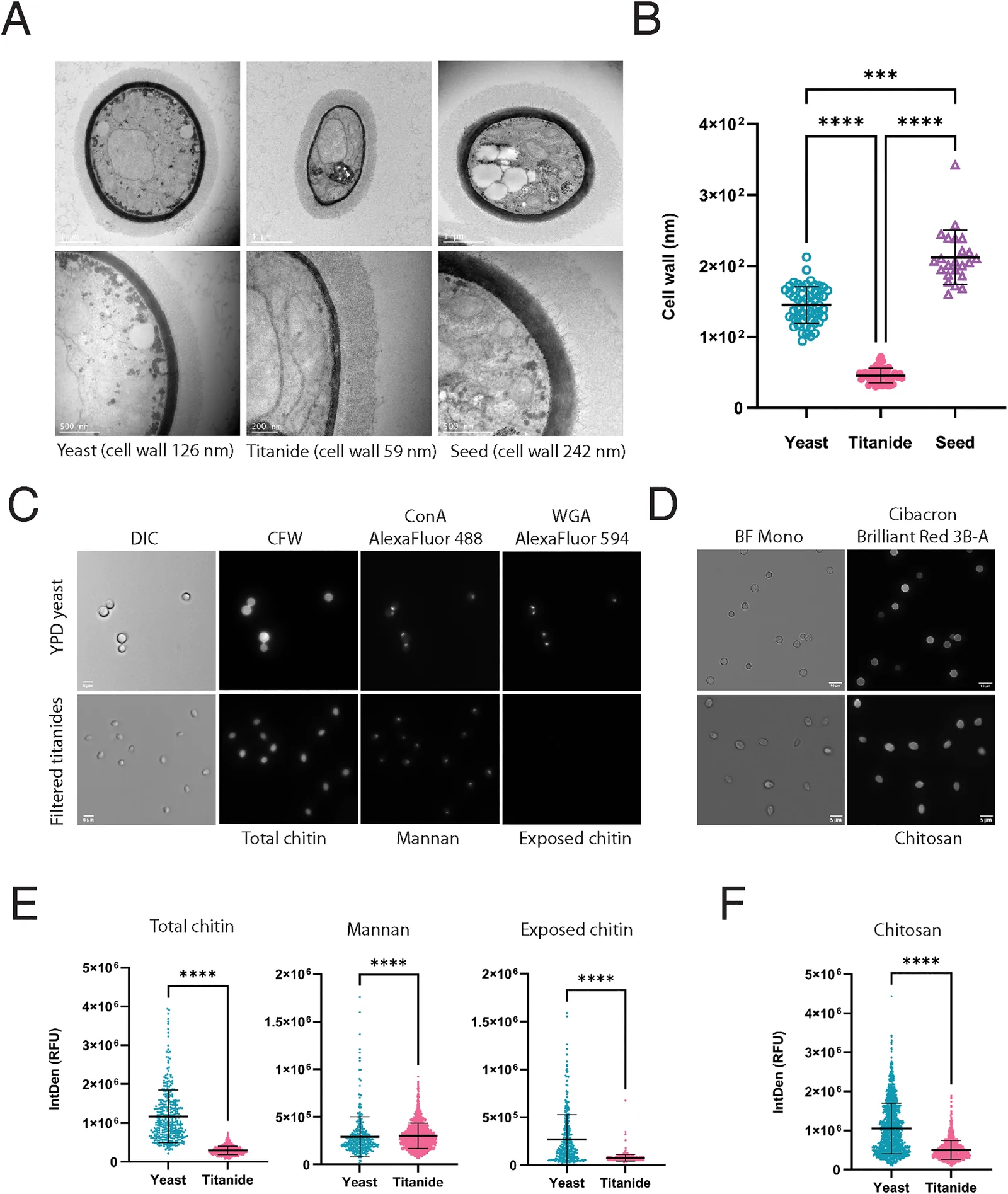
Titanides are distinct from yeast and seed cells in diameter and cell wall component exposure. **(A)** Representative TEM micrographs of YPD yeast, titanides, and seed cells. **(B)** Cell wall measurements (nm). ‘****’, *p* = <0.0001, ‘***’, *p* = 0.0006, assessed by Kruskal–Wallis with Dunnett’s multiple comparisons test. *n*= > 20. **(C)** Representative images of YPD-grown yeast (top) and titanides (bottom) stained for total chitin (CFW, left), mannan (ConA-AlexaFluor 488, middle), and exposed chitin (WGA-AlexaFluor 594, right). Imaged with 40× objective. Scale bar 5 µm. **(D)** Representative YPD-grown yeast (top) and titanides (bottom) stained for chitosan (Cibacron Brilliant Red 3B-A). Imaged at 40× objective. Yeast scale bar = 10 µm. Titanide scale bar = 5 µm. **(E)** Cells from (C) were quantified for Integrated Density (RFU). ‘****’, *p* = <0.0001, assessed by Mann–Whitney test. **(F)** Cells from (D) were quantified for Integrated Density (RFU). ‘****’, *p* = <0.0001, assessed by Mann–Whitney test. For all graphs, all cells in a given image were measured, with 3 images per sample, in biological triplicate, and mean and standard deviation indicated. The data underlying this figure can be found at https://doi.org/10.6084/m9.figshare.31171789.

The fungal cell wall is dynamic, changing significantly during morphological transitions, and is a defining feature of different *C. neoformans* morphotypes, including titan, typical, and seed cells [2,7,9,15,26]. We assessed the exposure of cell wall components of filtered 72-hour titanides compared to log-phase YPD-grown yeast, staining for total and exposed chitin, mannan, and chitosan, and observed differential localisation patterns of the major cell wall components (Figs 2C, 2D, and S3). Wheat Germ Agglutinin (WGA) reveals exposed chitin and Concanavalin A (ConA) binds mannose on mannoproteins in the outer cell wall (Figs 2C and S3). Cibacron 4 reveals chitosan, the deacetylated form of chitin and a key component of the *Cryptococcus* cell wall (Fig 2D) [27]. Titanide cells displayed single foci of mannan and exposed chitin, compared to dual foci on YPD-grown yeast, indicative of birth and bud scars (Figs 2C and S3). Quantification (Mean Integrated Density) revealed that titanides possess a significantly lower exposure of total chitin, exposed chitin, and chitosan (*p* = <0.0001 for all), but significantly higher exposure of mannan at the birth scar relative to YPD yeast (*p* = <0.0001) (Figs 2E and 2F).

Morphological switching in *C. neoformans* is well documented to alter polysaccharide capsule [4,5,15,28]. Capsule width was assessed for all cells in induced 72-hour cultures using two different monoclonal antibodies (mAb), 18b7 IgG, a canonical marker of total capsule that binds to an epitope in glucuronoxylomannan (GXM), the predominant constituent of capsule, and Crp127 IgM, which recognises an O-acetylated moiety of GXM (Figs 3A and S4) [28–30]. Titanides had significantly reduced capsule diameter compared to co-cultured typical and titan cells (*p* = <0.0001), with an average diameter of 1.3 ± 0.5 µm (Fig 3B and 3C). Staining further differentiated morphological subtypes based on epitope availability (Fig 3A, 3D, and S3). Typical cells exhibited a wide range of capsule staining intensities, reflecting heterogeneity in the availability of the 18b7 GXM epitope (Fig 3A and 3D). In contrast, taking cell size into account, titanides have more uniform and significantly lower epitope binding relative to co-cultured typical and titan cells (*p* = <0.0001) (Figs 3D and S4). Moreover, we observed mAb 18b7 staining predominantly throughout the titanide capsule, with a high intensity, suggesting altered distribution of the epitope (Fig 3A and 3B).

**Fig 3:**
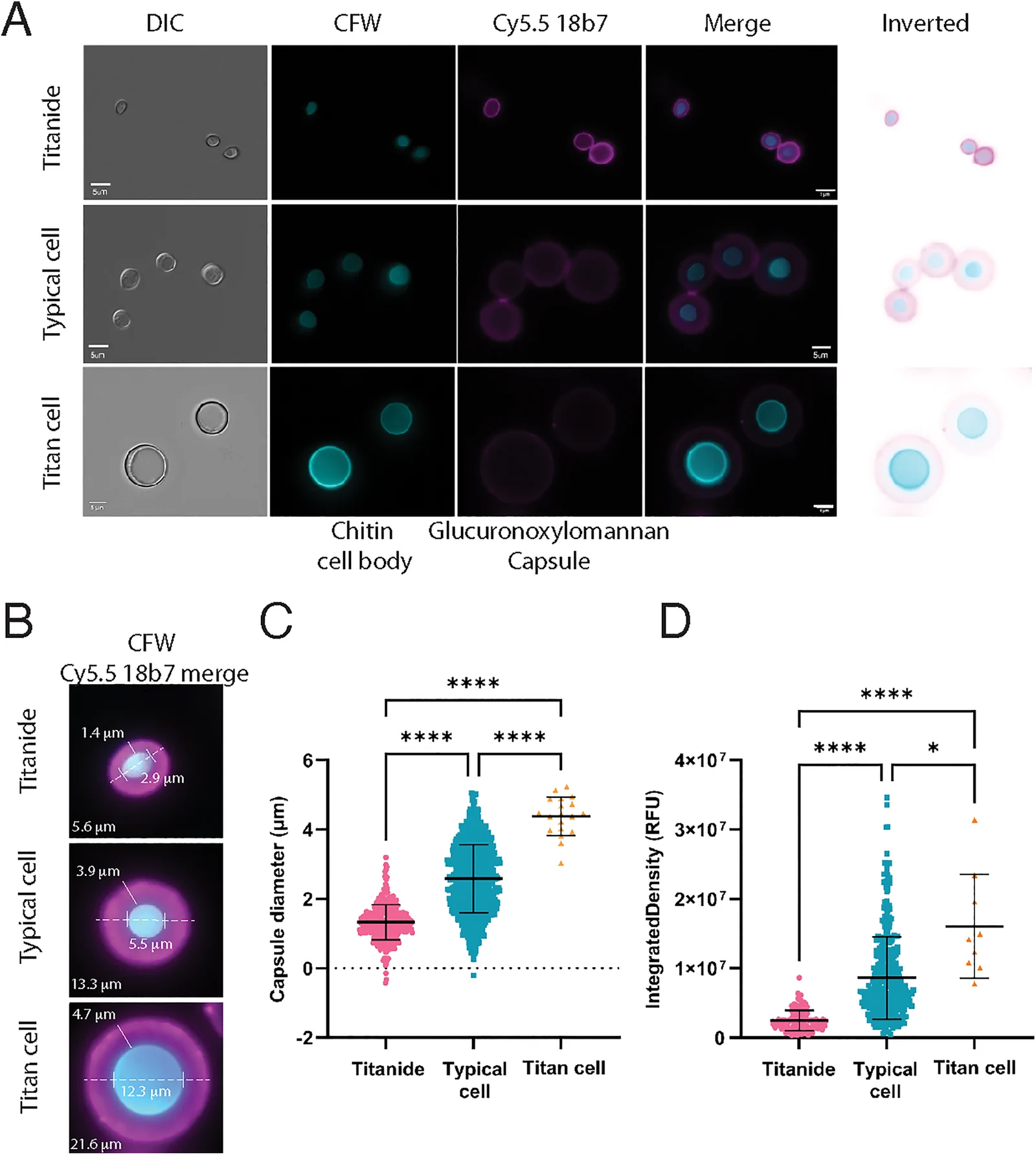
Titanides are distinct from typical and titan cells in capsule composition and diameter. **(A)** Representative images of filtered titanide (top panel), typical (middle), and titan cells (bottom). All cells were stained for the cell body (CFW, cyan) and GXM to visualise capsule size (mAb 18b7-Cy5.5, magenta). Inverted image also provided for contrast. Scale bar 5 µm. **(B)** Representative images and illustration of measurements for titanide, typical, and titan cells from **(A)**. **(C)** Cells from (A) were assessed for capsule thickness, calculated as (total capsule diameter-cell body diameter)/ 2. **(D)** Cells from (A) were categorised by size and quantified for mAb 18b7-Cy5.5 Integrated Density (RFU). For all capsule graphs, ‘****’, *p* = <0.0001, ‘*’, *p* = 0.0227, assessed by Kruskal–Wallis with Dunn’s multiple comparisons test. The data underlying this figure can be found at https://doi.org/10.6084/m9.figshare.31171789.

When titanides were compared directly to the other morphological subtypes by staining the total heterogenous populations with CFW, WGA, ConA, and Cibacron 4, we observed differential exposure of all major cell wall components (Fig 4A and 4B). Quantification revealed that, within this population, titanides possess significantly reduced total chitin, exposed chitin, mannan, and chitosan exposure compared to matched typical and titan cell counterparts (*p* = <0.0001 for all) (Fig 4C and 4D). Moreover, titanides again display a single birth scar, whereas typical and titan cells displayed dual or triple foci of ConA and WGA fluorescence consistent with bipolar birth and bud scars (Figs 2C and 4A).

**Fig 4:**
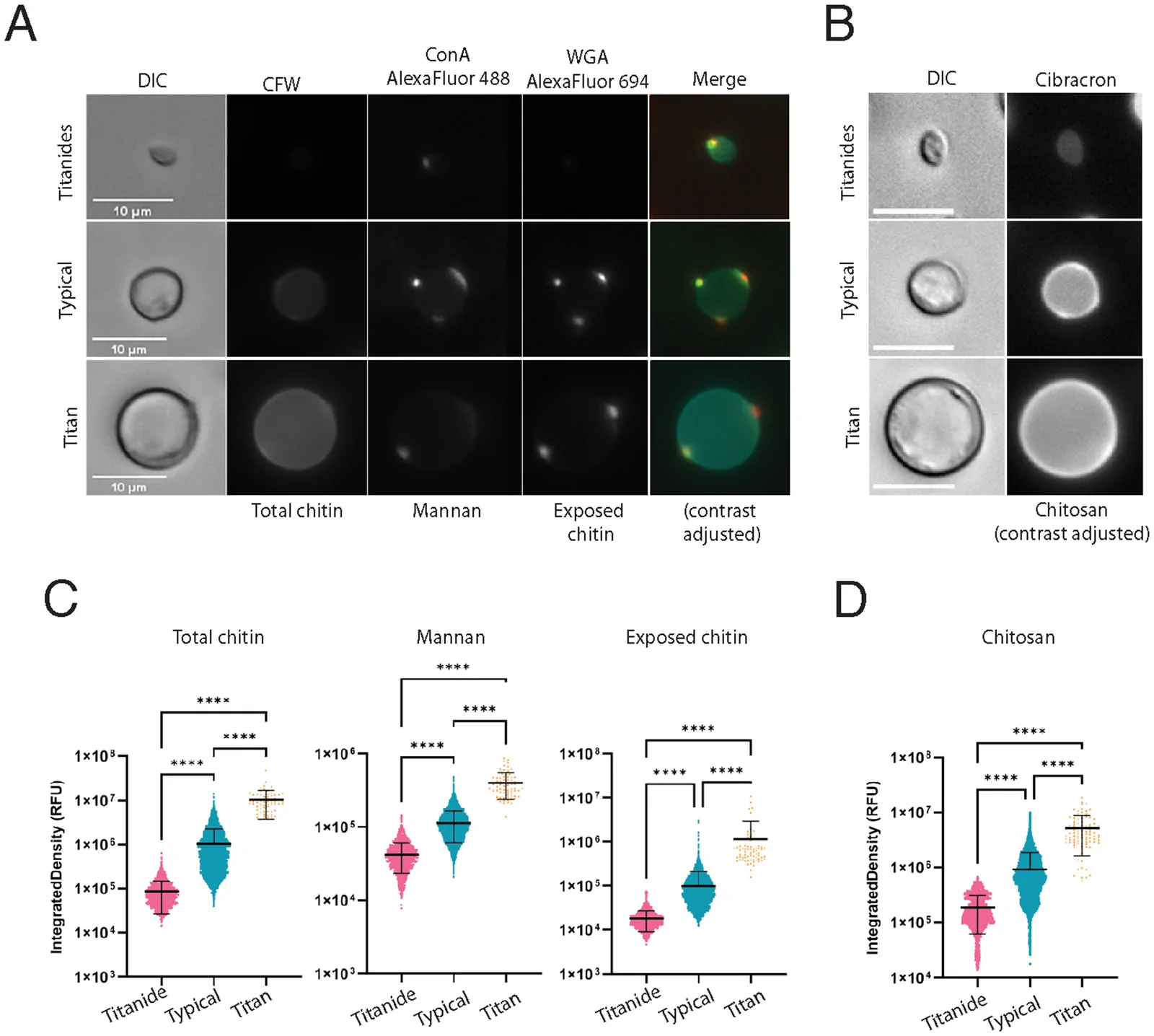
Titanides have distinct cell wall properties to typical and titan cells. **(A)** Representative images of a titanide (top panel), typical cell (middle), and titan cell (bottom) stained for total chitin (CFW, left), mannan (ConA-AlexaFluor 488, middle), and exposed chitin (WGA-AlexaFluor 594, right). Imaged with 40× objective. Scale bar 10 µm. **(B)** Representative images of a titanide (top panel), typical cell (middle), and titan cell (bottom) stained for chitosan (Cibacron Brilliant Red 3B-A). Imaged at 40× objective. Contrast adjusted for presentation only. Scale bar 10 µm. **(C)** Cells from (A) were quantified for Integrated Density (RFU). ‘****’, *p* = < 0.0001, assessed by Kruskal–Wallis with Dunn’s multiple comparisons test. **(D)** Cells from (B) were quantified for Integrated Density (RFU). ‘****’, *p* = < 0.0001, assessed by Kruskal–Wallis with Dunn’s multiple comparisons test. For all graphs, all cells were counted in a given image for 3 images, done in biological triplicate. The data underlying this figure can be found at https://doi.org/10.6084/m9.figshare.31171789.

Based on these characteristics, we morphologically define titanides as 2–4 µm oval cells with thin cell walls, reduced exposure of key cell wall components, single birth scars, and small capsules with altered GXM elaboration compared to typical and titan cells. Identification of these differences allows for a more detailed profile of titanides to emerge and raises hypotheses regarding growth and host interactions.

### Titanides are prevalent across the *C. neoformans* species complex

To assess the wider relevance of the titanide cell type, we examined the capacity of clinical and environmental isolates and related species to produce titanides in vitro. We screened 81 clinical and environmental isolates of the *Cryptococcus neoformans* species complex including subtypes VNIa, VNIb (the H99 subtype), VNBII, VNII, VNIII (hybrid), and VNIV (*C. deneoformans)*, for titanide formation after 72 hours and assessed for the presence or absence of titanides (S5A Fig). Titanide formation was observed across all 6 *C. neoformans* subtypes, and the vast majority of isolates generated titanides (74/81) (S5A and 5A Figs).

Cell size distribution analysis of a subset of 40 isolates representative of all VN types revealed that all *C. neoformans* isolates produced highly heterogeneous populations, but that the different morphological subtypes comprise different proportions of the total population across isolates (Fig 5B). Under these conditions after 72 hours, titanide frequency ranged from <1% to 50% of the population (Fig 5A and 5B). Typical cells comprised between 30 and 99% of the population (Fig 5A and 5B). Titan cells were rare, comprising <5% of the population across isolates; however, in many cases we observed cells that did not meet the >10 µm threshold for titan cells, but did observe cells with thickened cell walls and vacuoles consistent with the titan cell morphotype (“titan-like”) (Fig 5A and 5B). Overall, this highlights the overall population heterogeneity typical of *Cryptococcus* growth [5–7,11,15,23–25,31].

**Fig 5:**
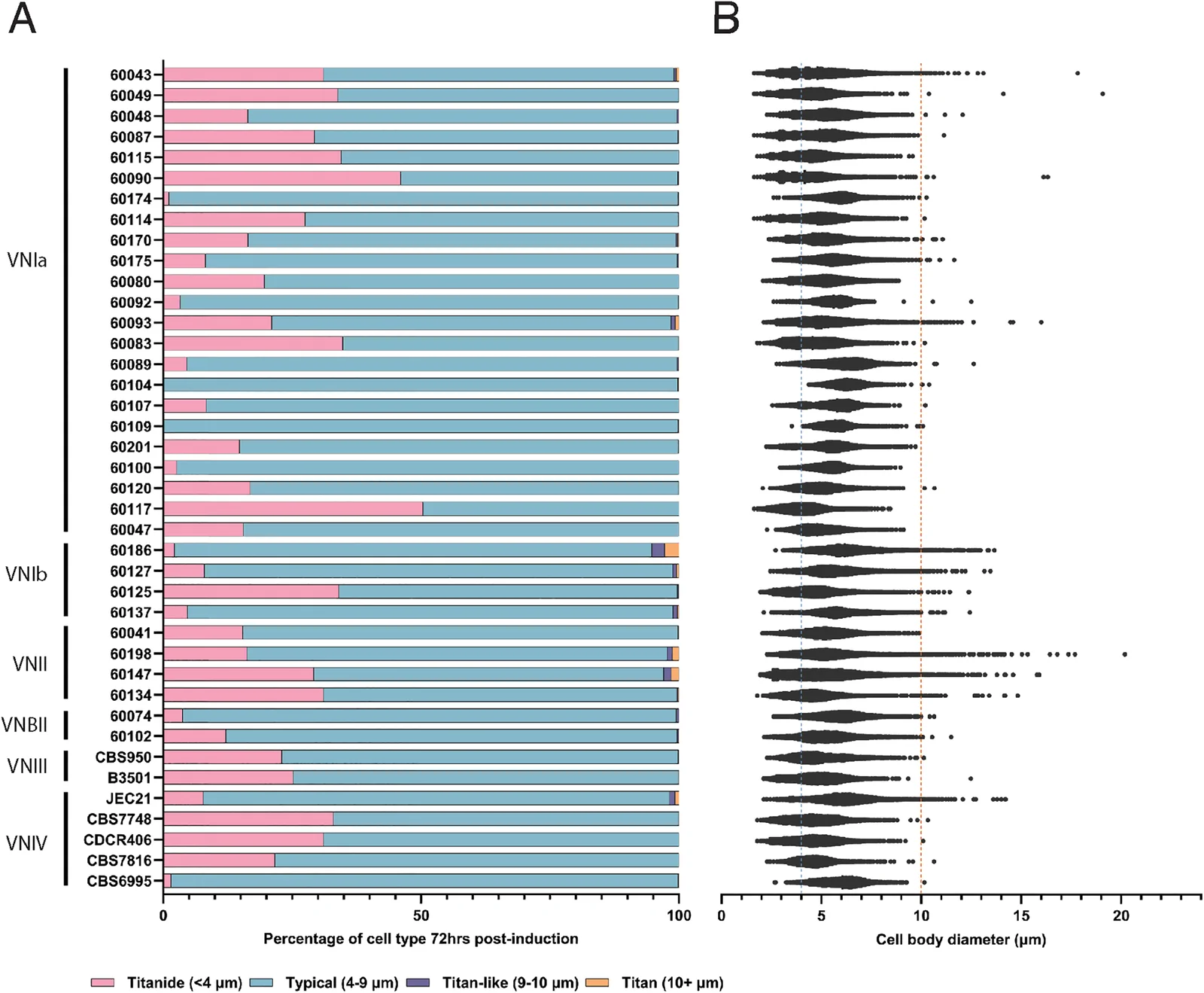
Clinical and environmental isolates across the *C. neoformans* species complex exhibit cellular heterogeneity, including titanides. **(A)** Percentage of titanide, typical, and titan cells in clinical and environmental isolates representing 6 *C. neoformans* subtypes 72 hours post-titan induction. **(B)** Cell diameter measurements for each isolate from **(A)**. Induced in biological triplicate. Measurements are representative of one biological replicate. Dotted line indicates 4 µm titanide cutoff and 10 µm titan cutoff. These data are a subset of those in S5A Fig. The data underlying this figure can be found at https://doi.org/10.6084/m9.figshare.31171789.

Finally, we additionally tested 8 *C. gattii* and 4 *Kwoniella* species isolates for capacity to produce titanides (S5 Fig). Inductions were performed using our standard conditions except that *Kwoniella* isolates, which are not tolerant to higher temperatures, were incubated at 30 °C. We observed no evidence of titanide formation in *C. gattii* or *Kwoniella* cultures (S5A Fig). It has previously been reported that the *C. neoformans/C. gattii* species complex is unique in the ability to undergo titanisation [32]. Consistent with this, no enlargement was observed in *Kwoniella* isolates at 72 hours under titan-inducing conditions. However, we did observe morphological heterogeneity in the form of hyphal structures produced by *Kwoniella pini* isolate CBS10737 (S5B Fig). Overall, this data suggests that titanide morphogenesis is specific to *C. neoformans* and is a conserved phenotype across lineages, but individual isolates exhibit different propensities to generate titanides.

Together, these data confirm the existence of oval 2–4 µm thin-walled titanides as a prevalent *C. neoformans* cell type. Due to their striking abundance across subtypes and isolates, the appearance in the literature of cells consistent with titanides in human lung histology, and the significance of morphogenesis for pathogenesis, we further investigated and characterised the biological potential of the titanide cell type [2,4,5,7,11,21,24,25,31]

### Titanides are morphologically plastic in different conditions

Under inducing conditions, population growth occurs via proliferation of typical and titan cells (S1 Movie), with typical cells converting through endoreduplication into titan cells, and titan cells producing small daughter cells with the capacity to again produce titan cells [2]. To understand the contribution of titanides to this process, filtered titanides were stained with CFW and inoculated into fresh inducing medium. Titanide inocula regenerated heterogenous cultures of titan and typical cells within 24 hours (Fig 6A).

**Fig 6:**
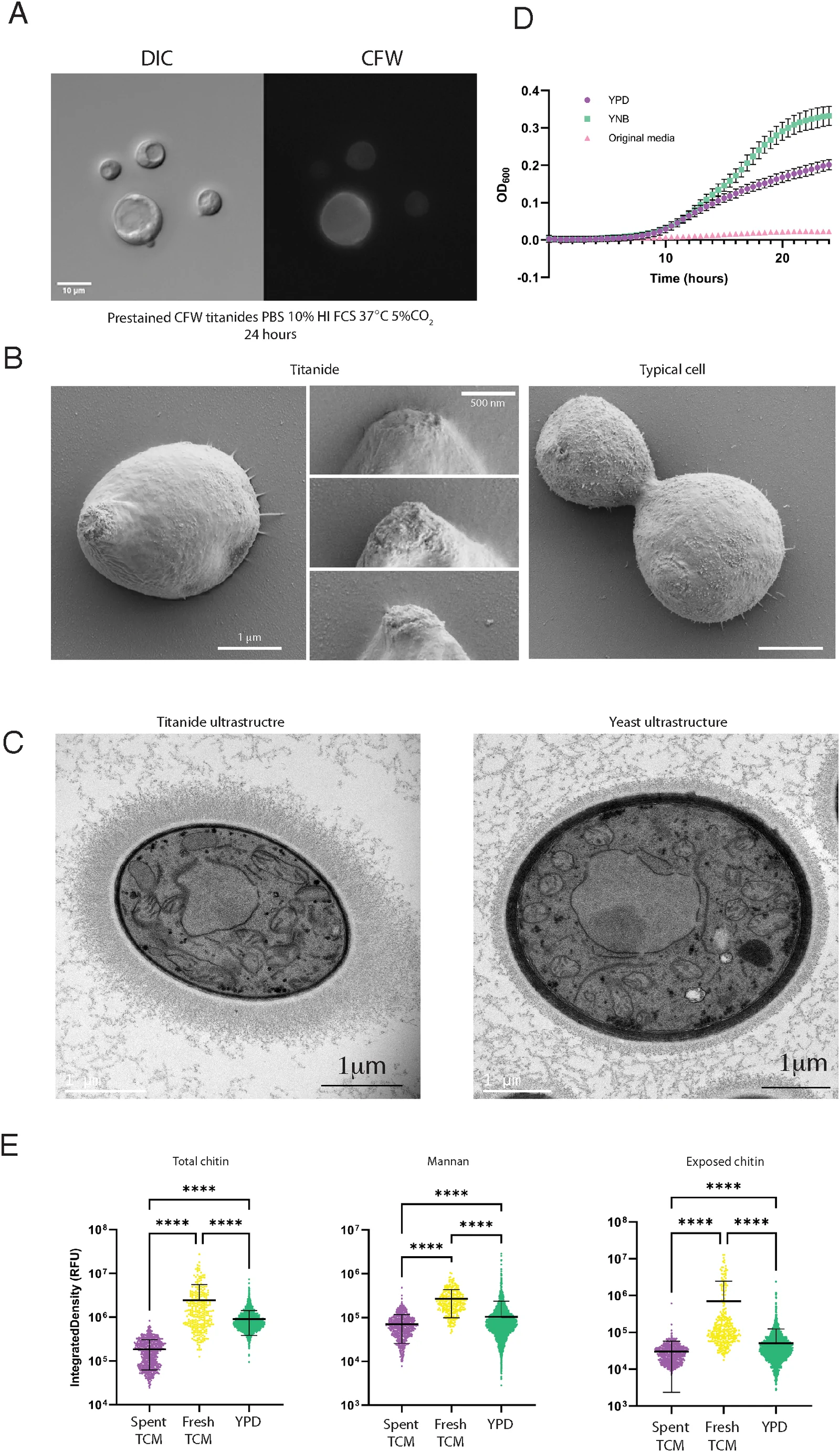
Titanide morphology and cell wall composition changes following growth in new media. **(A)** Filtered titanides were pre-stained with CFW and incubated in fresh inducing conditions for 24 hours. Imaging revealed reconstitution of the heterogeneous population. Scale bar 10 µm. **(B)** Representative images of titanide and typical cell birth/bud scars. Cells treated with DMSO to reduce capsule. Scale bar 1 µm, zoomed in scale bar 500 nm. **(C)** Representative TEM micrographs of titanide and yeast ultrastructure. Scale bar 1 µm. **(D)** Automated growth curves of filtered titanides inoculated into liquid YPD, YNB 2% glucose, and the media from which they were cultured (72-hour conditioned media) at 30 °C. Error bars represent SD of the three biological replicates. **(E)** Quantification of cell wall composition for titanides cultured for 24 hours under the indicated conditions. Integrated Density (RFU) is reported for CFW, ConA, and WGA fluorescence. All cells were counted in a given image for 3 images, done in biological triplicate. Mean indicated by the black line. ‘****’, *p* = <0.0001, assessed by Kruskal–Wallis with Dunnett’s multiple comparisons test. Values correspond to cultures shown in S6 Fig. The data underlying this figure can be found at https://doi.org/10.6084/m9.figshare.31171789.

Staining with CFW and WGA suggested that titanides exhibit a single birth scar, and no bud scars (Figs 2C and 4A). To rule out that titanides bud adjacent to their birth scar, we performed SEM on total induced populations (Fig 6B). Cells were pre-treated with DMSO to reduce capsule and allow observation of the cell surface. On titanides, SEM revealed the presence of a single birth scar, whereas typical cells revealed differentiated birth and bud scars (Fig 6B). This suggests that titanides may not be proliferative. However, we previously showed by TEM ultrastructure imaging of mixed cultures that thin-walled oval cells contained organelles and sub-cellular structures consistent with the potential for metabolic activity, including ribosomes, mitochondria, and a nucleus, and repeated TEM imaging using 72-hour filtered populations confirmed this observation (Fig 6C) [2].

To directly observe titanide cell proliferative capacity, we performed growth curve and culture analysis of filtered populations incubated in their original media at 30 °C. This revealed that titanides are non-proliferative under this condition (Figs 6D and S6). However, when purified titanide cultures were transferred to either rich YPD medium or Yeast Nitrogen Base (YNB) medium at 30 °C, proliferation was restored (Fig 6D). Microscopy of titanides incubated in YPD revealed a uniform typical cell population (S6A and S6B Fig). Analysis of size and cell wall properties of titanides in their original media, fresh inducing media, or YPD for 24 hours revealed changes consistent with reconstitution of the heterogenous population or uniform yeast populations, respectively (Figs 6E, S6A, and S6B). Overall, this demonstrates that titanide populations can reconstitute the different cell morphotypes, including heterogenous host-relevant populations and homogenous yeast populations. Taken together these data demonstrate that growth condition, and not starting cell type, influences population morphology.

### Titanides resume growth synchronously, accompanied by release of nuclear compaction

Interestingly, we observed a 10-hour lag phase before titanides resumed proliferation (Fig 6D). To better understand this delay, we performed microfluidic-assisted live cell imaging of titanide growth. This revealed that titanides exhibit highly synchronous behaviour. As can be seen in the timelapse montage in Fig 7A, titanides incubated in YPD medium exhibit three stages of growth: (i) a period of dormancy lasting ~5 hours; (ii) progenitor titanide cells gradually swell over 300 min from an average of 2.57 μm up to 4.67 μm in diameter; (iii) swollen cell size then plateaus. Cells proliferate synchronously to produce typical-sized daughters (Fig 7A and 7B; S2 Movie). Quantification of cell size over time revealed a consistent rate of swelling of 0.0069 µm/min (Fig 7C).

**Fig 7:**
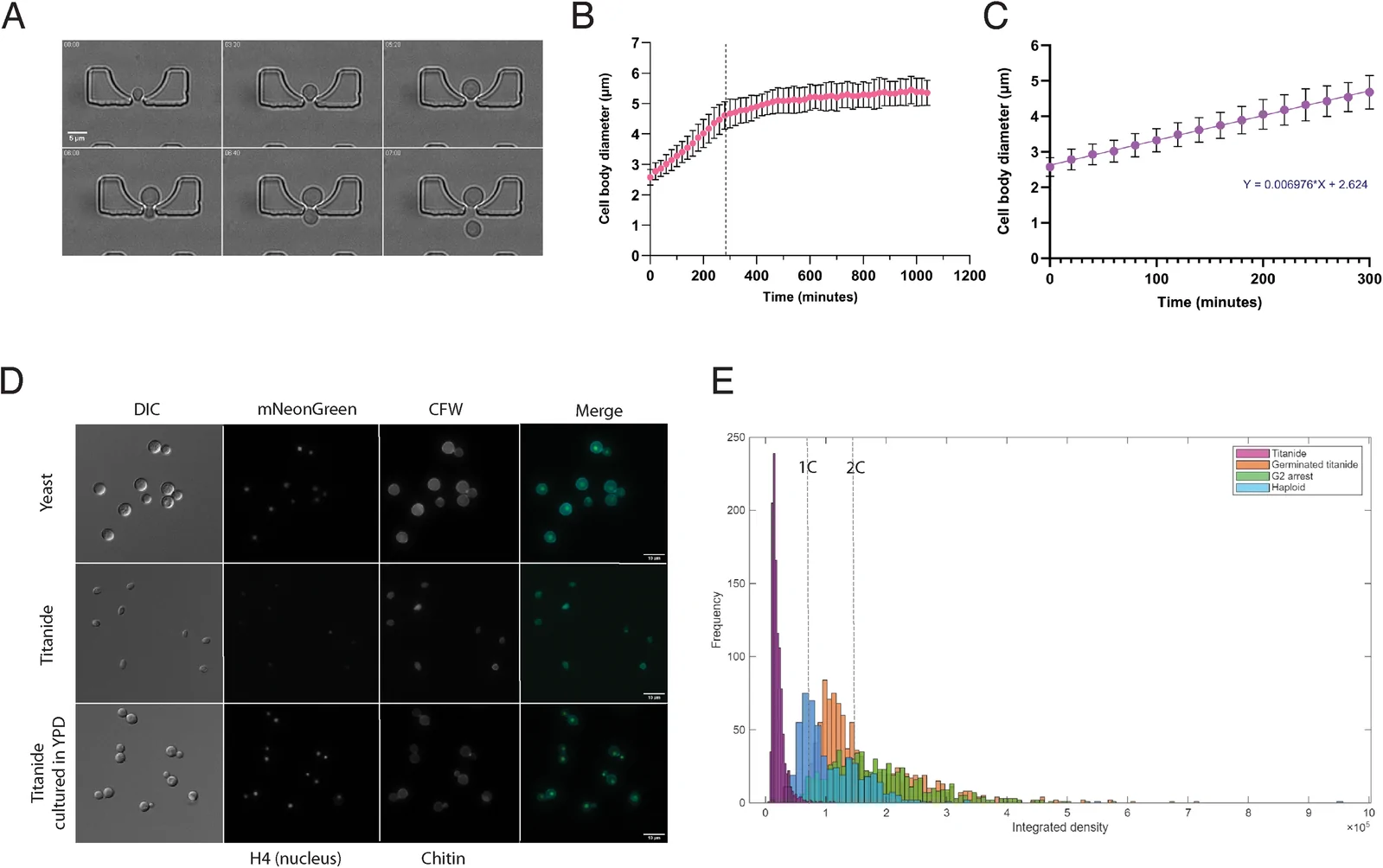
Titanides are non-proliferative in their original media but swell synchronously as yeast in fresh media. **(A)** Representative montage from microfluidic-assisted live imaging of titanide growth in YPD media. Full time series is presented in S2 Movie. **(B)** Representative measurements from 1 biological replicate of cell body diameter plotted against time. Error bars represent single-cell measurements across three FOV. Dotted line represents average time of daughter production during swelling. **(C)** Linear regression of cell body diameter plotted against time for titanides in the first 300 min in rich media before cell size plateaus to determine swelling rate during this period for two biological replicates. Swelling rate was calculated based on 2 biological replicates (161 cells) as the slope of the line on the diameter-time graph. Error bars represent single-cell measurements across three FOV. **(D)** Representative images of histone-4 tagged mNeonGreen reporter strain haploid YPD yeast (top), 72-hour titanides (middle), and swollen titanides (bottom) stained with CFW to visualise cell body. Scale bar 10 µm. **(E)** Cells from (D) alongside G2-arrested control were quantified for Integrated Density (RFU) and data visualised as a histogram to identify 1C and 2C peaks (indicated by dotted lines) and assess genome content of titanides pre- and post-swelling. All cells in a given image were counted for 3 images, in biological triplicate. The data underlying this figure can be found at https://doi.org/10.6084/m9.figshare.31171789.

Once in a state of active growth, titanide-derived yeast were highly proliferative relative to a typical cell control grown under the same conditions, with a mean replication time of 59.4 min compared to 70.6 min (S3 Movie).

We previously showed by flow cytometry that induced cultures exhibit altered DNA staining patterns, revealing a population of cells with apparent “sub-1C” DNA content, similar to ungerminated spores [18]. Because titanides do not exhibit reduced viability, we hypothesised that ‘sub-1C’ staining may be due to changes in DNA compaction preventing dye intercalation. To better understand the dynamics of titanide DNA compaction, we used a strain in which histone H4 is tagged with mNeonGreen (Fig 7D). This strain demonstrated only mild sensitivity to genotoxic stress, suggesting the tag does not disrupt chromatin organisation (S7A Fig). For YPD-grown haploid and G2-arrested cells, we could identify fluorescence intensity consistent with 1C and 2C DNA content using this strain (Fig 7E). Moreover, we observed that titanides have low fluorescence compared to these controls (Figs 7D, 7E, and S8), consistent with a ‘sub-1C’ DNA content. However, upon release into YPD media, synchronously proliferating titanides exhibited H4 levels consistent with S-phase DNA synthesis, with no evidence of reduced DNA content (Fig 7E). No difference in sensitivity to genotoxic stress (TBZ) was observed for any of these cell types (S7B Fig). Together, these data suggest that following birth, titanides are arrested with nuclei in a compacted state that is released upon reactivation and proliferation. This process, and the nuclear changes that accompany it, will be the subject of future work.

### Typical cells, not titan cells, are the titanide progenitor

Because titanides are associated with titan-inducing conditions, and titan cells proliferate asymmetrically, producing small daughter cells, it has been postulated that titanides are the progeny of titan mothers [7,31]. Interestingly, in our screen of clinical isolates, we identified strains that did not titanise, but titanides were still present, in addition to strains that did form titan cells but did not generate titanide daughters (Fig 5A and 5B). To better understand the exact origin of titanides, we performed microfluidics-assisted live cell imaging of proliferation under inducing conditions using microfluidic traps. For this analysis, we selected KN99, as we observed earlier formation (6 versus 7 hours) and a higher percentage of titanides at 24 hours (9.27% versus 3.98%) compared to H99, increasing chances of capturing titanide birth (Figs 1F, S1A, and S1B).

To test the hypothesis that titanides were the daughters of titan mothers, titan cultures were pre-induced for 48 hours under standard conditions, then filtered to enrich for titan cells. Cells were loaded onto traps, then imaged under continuous perfusion with 10% FCS in PBS for 24 hours. Titan mothers budded asymmetrically, consistently producing daughter cells that were round and ~4 µm in diameter (S4 Movie). Additionally, titan daughters rapidly enter a proliferative cell cycle and divide as typical cells (S4 Movie). This suggests that titan cells are not the progenitor cells for titanides.

To further understand titanide origins, we imaged total populations using microfluidic traps to follow the entire population and identify the emergence of our cell of interest. KN99 cells were seeded into microfluidic traps under continuous perfusion with 10% FCS in PBS, 37 °C, 5% CO_2_ for 48 hours, and then imaged for 4 hours. Using this population, we observed daughters matching our established titanide definition of oval 2–4 µm, low CFW, non-proliferative cells within 30 min, and titanides continued to emerge across the field of view for the duration of imaging (4 hours) (Fig 8A; S5 Movie). Size measurement of progenitor cells that produced titanides revealed an average cell size of 4.9 µm, consistent with a typical mother, not titan mother, progenitor for titanides (Fig 8A and 8B).

**Fig 8:**
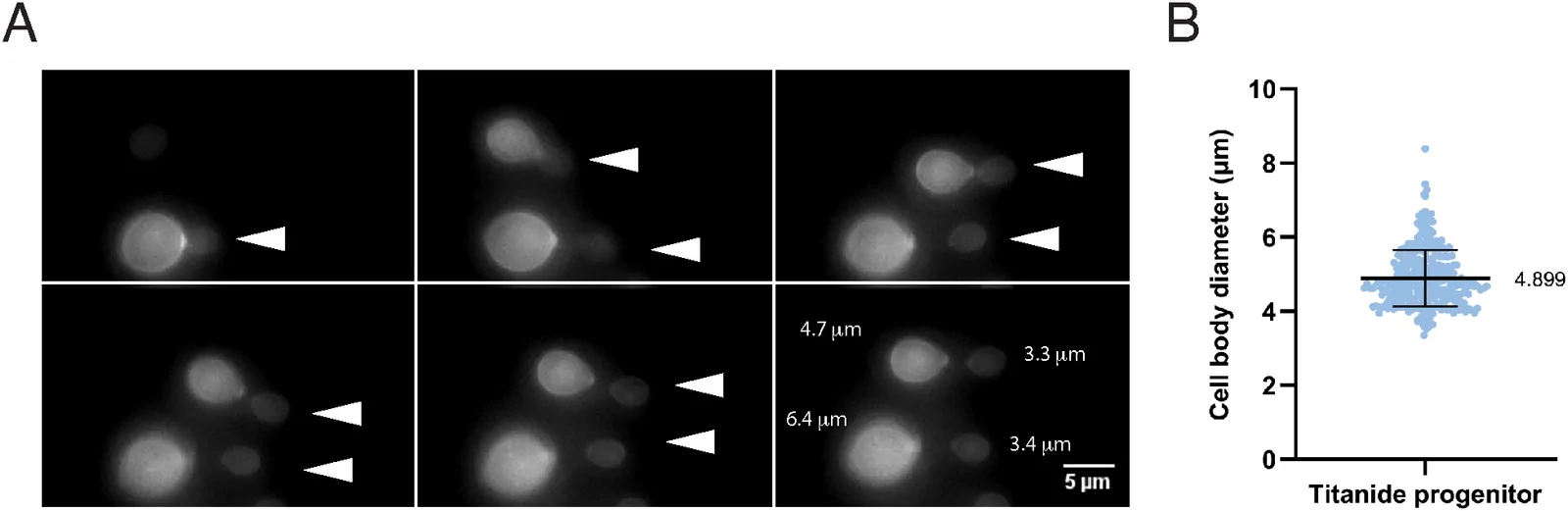
Titanides are the progeny of typical cells. **(A)** Representative montage from microfluidic-assisted time-lapse imaging of typical cells under inducing conditions and stained for total chitin (CFW). Titanide daughters are indicated by arrow heads. Full time series is presented in S5 Movie. Scale bar 5 µm. **(B)** Diameter of progenitors that produced titanide daughters. 3 biological replicates, with mean and SD indicated. The data underlying this figure can be found at https://doi.org/10.6084/m9.figshare.31171789.

### Titanides are more resistant than yeast to some exogenous stressors

#### SDS stress impacts swelling, but not viability, of titanides.

To unravel the biological relevance of titanides, sorted populations were tested for sensitivity to a range of cell stressors, including compounds that impact cell wall integrity given the unique cell walls characteristic of titanides. Stresses included caffeine, to test the cell integrity (CWI pathway), SDS which disrupts the plasma membrane, and NaCl for osmotic stress resistance [33,34]. H_2_O_2_ and DPTA NONOate were also used to test for sensitivity to oxidative and nitrosative stress, respectively, as titan cells are highly resistant to this type of stress [4,5,19]. Cell growth was assessed in 96-well plates for 24 hours in YPD plus stress compounds to capture the impact of stress exposure on both the initial titanide form and the subsequent population of ‘titanide-derived’ yeast following swelling (Fig 9A and 9B).

**Fig 9:**
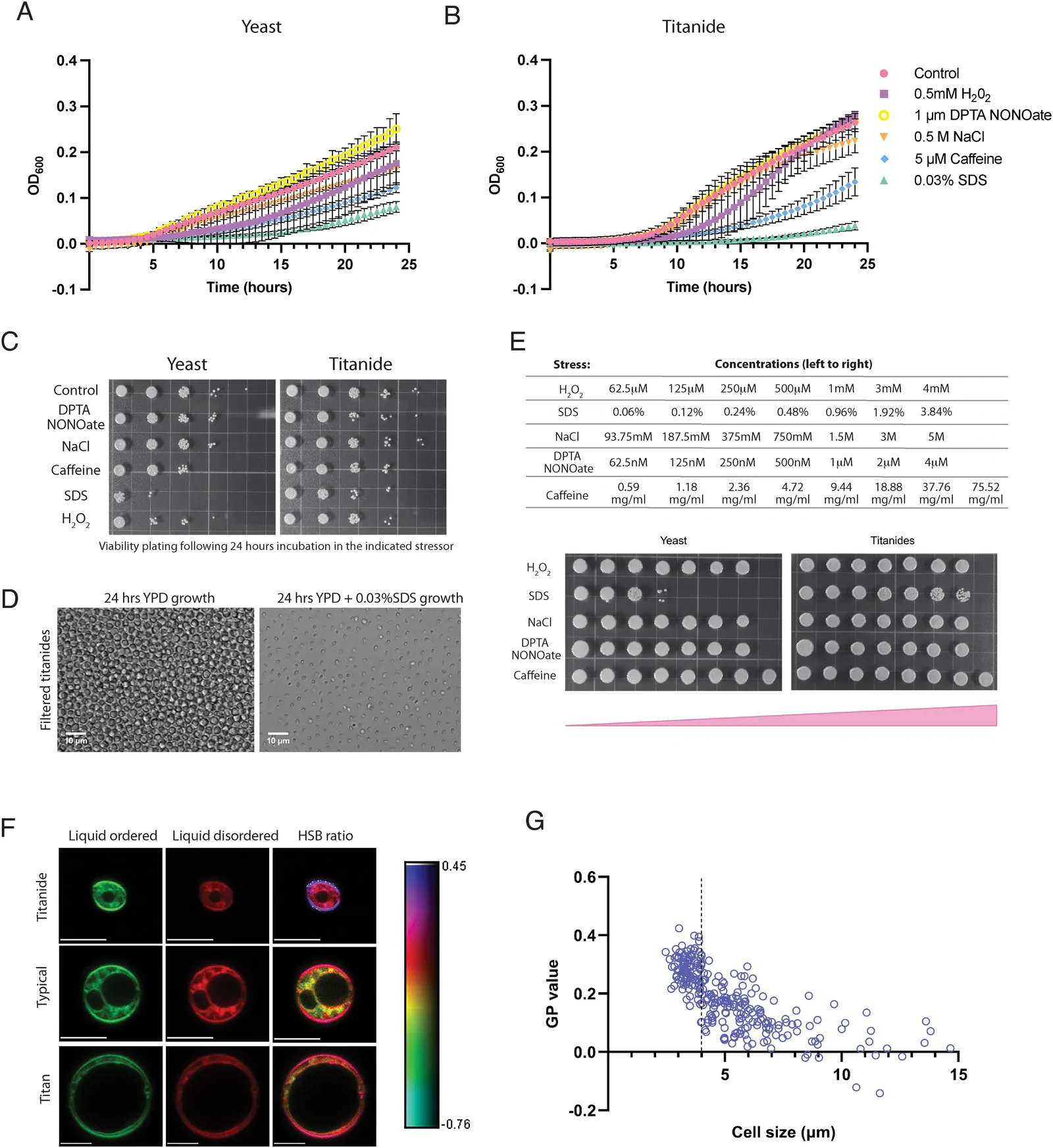
Titanide swelling rates, but not viability, are impacted by caffeine and SDS. **(A, B)** Growth curves of yeast (A) and filtered titanides (B) in liquid YPD containing sodium dodecyl sulphate (0.03%), hydrogen peroxide (0.5 mM), sodium chloride (0.5 **M)**, caffeine (10 µg/ml), and DPTA NONOate (1 µM). Error bars represent standard deviation of three biological replicates. **(C)** Serial dilutions of cells from (A) and (B) were plated onto YPD 2% glucose to assess viability following incubation in long-term stress. **(D)** Representative images of titanides following growth for 24 hours in YPD or YPD plus 0.03% SDS. Scale bar 10 µm. **(E)** Titanides display increased resistance to short-term SDS stress. Table of concentrations of selected cell stress agents in which YPD-grown yeast or titanide cells were incubated in (top) and YPD spot plates of cells plated following 30-min incubation in increasing concentrations of indicated cell stress reagents (bottom). **(F)** Representative single-plane liquid-ordered, disordered, and pseudo-coloured generalised polarisation (GP) images of a 48-hour titanide (top), typical (middle), and titan cell (bottom) following staining with the ratiometric dye di-4-ANEPPDHQ. LUT bar designates the range of GP values where white/navy shows high membrane order and greens/blues show low membrane order in pseudo-coloured images. Scale bar 5 μm. **(G)** Cells from (F) were quantified for cell diameter and mean GP value. Plasma membrane zones were used to quantify mean GP values. Dotted line represents 4 μm cut-off to highlight difference in membrane order of small cells within the heterogenous titan population. Done in biological triplicate. The data underlying this figure can be found at https://doi.org/10.6084/m9.figshare.31171789.

Interestingly, relative to YPD-grown yeast, we observed no difference in the sensitivity profiles of ‘titanide-derived’ yeast, except for a modest ability to better resist H_2_O_2_ (Fig 9A and 9B). For both groups, the biggest effects were seen in response to caffeine and SDS, however, the two cell types responded differently (Fig 9A and 9B). Yeast cells were less viable after growth for 24 hours (Fig 9C). In contrast, for ‘titanide-derived’ yeast, the stressors impacted initial swelling but not viability (Fig 9C). Caffeine stress was associated with slower growth overall, while titanides exposed to SDS stress did not increase in OD. Inspection of the cells after 24 hours revealed persistence of distinct small, oval cells (Fig 9D). Furthermore, when SDS stress was removed and the titanide cells were plated onto YPD agar, we observed growth consistent with media-only grown cells (Fig 9C). This suggests that membrane perturbation disrupts the swelling process without killing titanides.

#### Titanides and titanide-derived yeast are more resistant to SDS stress than yeast cells.

To better understand the role of proliferation on titanide stress resistance to SDS and H_2_O_2_, we tested the impact of short-term stress. Purified titanides and YPD-grown yeast were incubated in increasing concentrations of the previously tested stressors for 30 min, then plated to determine viability (Fig 9E). This tests stress resistance while limiting the impact of proliferation on sensitivity. Differences were only detected between the cell types in response to SDS; however, this did not explain the moderate resistance to H_2_O_2_. To compare H_2_O_2_ resistance profiles in actively proliferating titanide-derived yeast to titanides themselves, pre-swollen titanides were subjected to H_2_O_2_ for 24 hours (S9A and S9B Fig). Relative to its relevant control, titanides exposed to stress for 24 hours have moderately higher end-point ODs than YPD yeast (S9C and S9D Fig). However, pre-swollen titanides lose this phenotype (S9C and S9D Fig). Interestingly, early on there does seem to be some increased resistance in titanide-derived yeast relative to YPD yeast, suggesting there may be some increased stress resistance in these cells that is lost over time (S9B Fig).

#### Titanides have a highly ordered plasma membrane.

As seen in Fig 9E, titanides still displayed an increased resistance to SDS stress compared to YPD-yeast, independent of proliferation. Titanides therefore appear to exhibit increased resistance to cell membrane stressors, despite their significantly thinner cell walls. To better understand this, we investigated the lipid membrane organisation of the different morphological subtypes. The polarity-sensitive ratiometric dye di-4-ANEPPDHQ gives a readout of lipid order and packing, based on spectral shifts upon dye penetration into the liquid disordered phase (Fig 9F) [35]. This can reflect differences in lipid saturation or composition [36]. Interestingly, membrane heterogeneity was observed under titan culture conditions (Fig 9F and 9G). Quantification revealed that in titanide cells (<4 µm), lipids in the plasma membrane were highly ordered, while typical and titan cells had lower lipid packing values (generalised polarisation, GP) (Fig 9F and 9G). These data suggest that titanide resistance to SDS may be due to membrane composition and organisation.

### Titanides are hypersensitive to Amphotericin B

A key component of the fungal cell membrane is ergosterol, and increased lipid order is associated with increased ergosterol incorporation [37]. Ergosterol is a major antifungal target; Therefore, MICs for antifungals that target ergosterol were assessed for titanides and yeast using an adaptation of established EUCAST protocols with microdilution plates. No differences in growth inhibition or viability was observed in response to fluconazole (MIC50 of 4 µg/ml) (Fig 10A). However, for Amphotericin B, while we observed the expected MIC of 0.25 µg/ml for yeast cells, titanides were hypersensitive, with 0.06 µg/ml inhibiting growth and significantly reducing viability (Fig 10A). Titanides were also more sensitive than YPD yeast to Nystatin, a polyene that binds directly to ergosterol in plasma membrane, with an MIC of 1 µg/ml, as opposed to 2 µg/ml for YPD-yeast [38].

**Fig 10:**
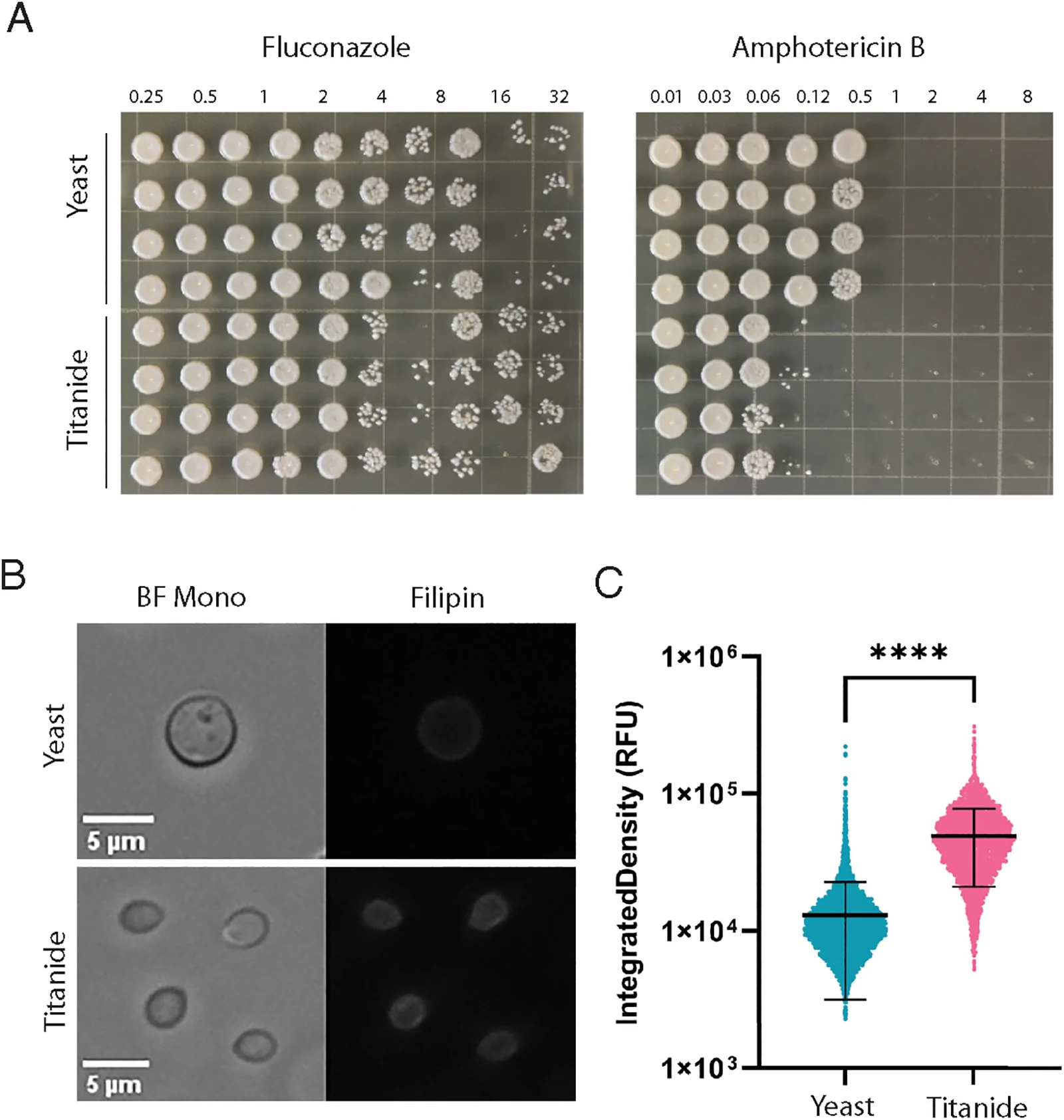
Titanides are hypersensitive to Amphotericin B. **(A)** Following MIC testing to fluconazole and amphotericin B, cells were plated onto YPD 2% glucose to determine fungicidal effect. All plates were incubated at 30 °C for 48 hours. **(B)** Representative images of YPD yeast (top) and titanides (bottom) stained for ergosterol content with Filipin. Scale bar 5 μm. **(C)** Cells from (B) were quantified for Integrated Density (RFU). ‘****’, *p* = <0.0001, assessed by Mann–Whitney *t t*est. All cells in a given image were measured, with 3 images per sample, in biological triplicate, and mean and standard deviation indicated. The data underlying this figure can be found at https://doi.org/10.6084/m9.figshare.31171789.

Polyenes act as sponges to rapidly extract ergosterol from the membrane, leading to membrane disruption and cell death [39]. Reduced ergosterol is associated with increased polyene resistance [39,40]. We used the filipin stain, which specifically binds to ergosterol, to quantify relative ergosterol levels in titanides compared to YPD-grown yeast (Fig 10B and 10C) [41,42]. Quantification revealed that titanides possess significantly more ergosterol relative to YPD-yeast (*p* = <0.001) (Fig 10C). Altogether, this is consistent with a model in which titanide hypersensitivity to polyenes is a result of high ergosterol content of these cells.

### Titanide interaction with phagocytic cells is dependent on opsonisation and is delayed relative to yeast cells

The *Cryptococcus* capsule is a key virulence factor that blocks recognition and uptake by phagocytes [43]. Given the differences in capsule and cell surface properties of titanides, we tested the capacity of murine-derived J774 macrophages to phagocytose titanides via timelapse microscopy (Fig 11A and 11B) [44]. It is well established that the *Cryptococcus* capsule prevents recognition and uptake by phagocytic cells by blocking the availability of key ligands on the cell wall (beta-glucan, chitin, mannan). Instead, *Cryptococcus* uptake relies on opsonisation by complement or antibodies (See [43,45] and [30]). First, we assessed uptake of un-opsonised yeast and titanides. Consistent with previous observations, phagocytosis of un-opsonised yeast was rarely observed, and un-opsonised titanide cells were also rarely phagocytosed likely due to the presence of capsule (S6A, S6B Movie) [46,47]. When cells were opsonised with mAb18b7, phagocytosis of both cell types by macrophages was observed within 30 min of incubation. Uptake events were quantified via live cell imaging, with an event counted each time a macrophage engulfed fungal cells (Fig 11A and 11B). Quantification was stopped after 2 hours to exclude the impact of yeast cell proliferation on uptake dynamics. We observed a slight but statistically significant reduction (71% versus 86%, *p* = <0.0001) in the number of macrophages engulfing titanides compared to YPD-grown yeast (Fig 11A and 11B; S7A and S7B Movie). Overall, titanides require opsonisation for uptake and are less preferentially phagocytosed by murine macrophages.

**Fig 11:**
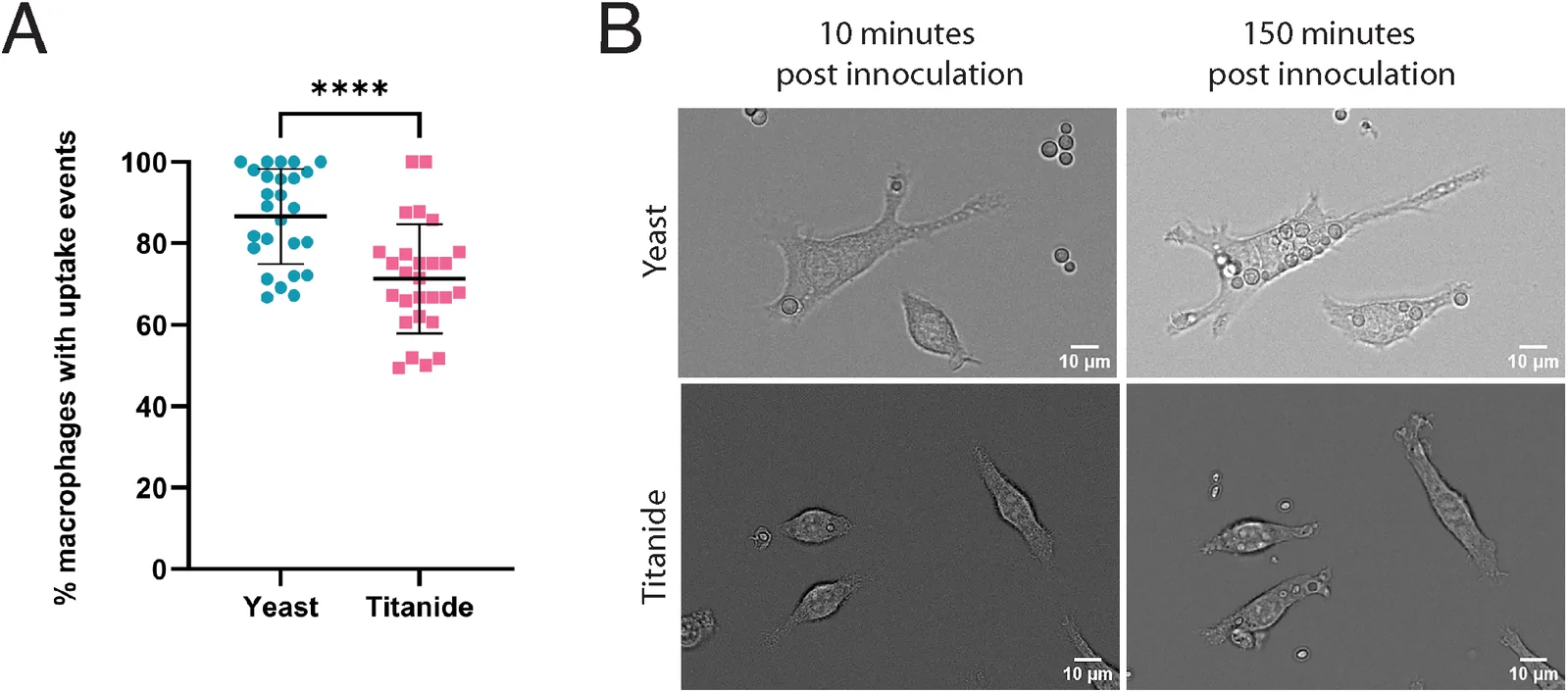
Titanides are less preferentially taken up by murine macrophages. **(A)** Percentage of J774 murine macrophages (stimulated with IFN-γ and LPS) with at least one uptake event of an opsonised *C. neoformans* YPD yeast or titanide across 2 hours co-incubation. ‘****’, *p* = <0.0001, assessed by Mann–Whitney *t t*est. Mean indicated by the black line. **(B)** Representative images of macrophages after 10 min and 150 min following co-incubation with YPD yeast (top) and titanide cells (bottom). Scale bar 10 µm. The data underlying this figure can be found at https://doi.org/10.6084/m9.figshare.31171789.

### Titanides are fully pathogenic in a murine inhalation model of disease

Particles greater than 5 µm are subject to efficient clearing by the upper respiratory tract and increased penetration into the lower airways may be achieved by infectious particles ≤2 µm [48,49]. Therefore, alongside studies demonstrating their virulence in a murine model, basidiospores have been the most accepted candidates for the infectious propagule of cryptococcal infection [47,48].

Due to the morphological similarities between titanides and spores (2–3 µm, oval), in addition to behavioural properties reminiscent of each other such as abundance, growth arrest until germination, and increased stress resistance to some stressors, we postulated that the *C. neoformans* titanide could also serve as an infectious propagule.

To gain insights into the fate of titanides during the early stages of infection, groups of 10 C57BL/6 black mice were infected by intranasal inoculation with titanides or YPD-grown rich yeast derived from H99 *C. neoformans* strain [50]. CFU analysis was performed at days 3 and 5 post-infection (3 mice per group). Titanides displayed no differences in ability to colonise the lung as an infectious propagule, with no significant differences in CFU recovered from titanide and yeast-infected mice at days 3 and 5 post-infection (*p* = 0.6243 3 dpi and *p* = 0.1224 5 dpi) (Fig 12A). Histological analysis revealed that, consistent with our in vitro observations, following infection with titanides fungal populations within the lungs were heterogenous, with titanide, typical, and titan cells, present, typical of *C. neoformans* infection at days 3 and 5 (Figs 12B and S10B).

**Fig 12:**
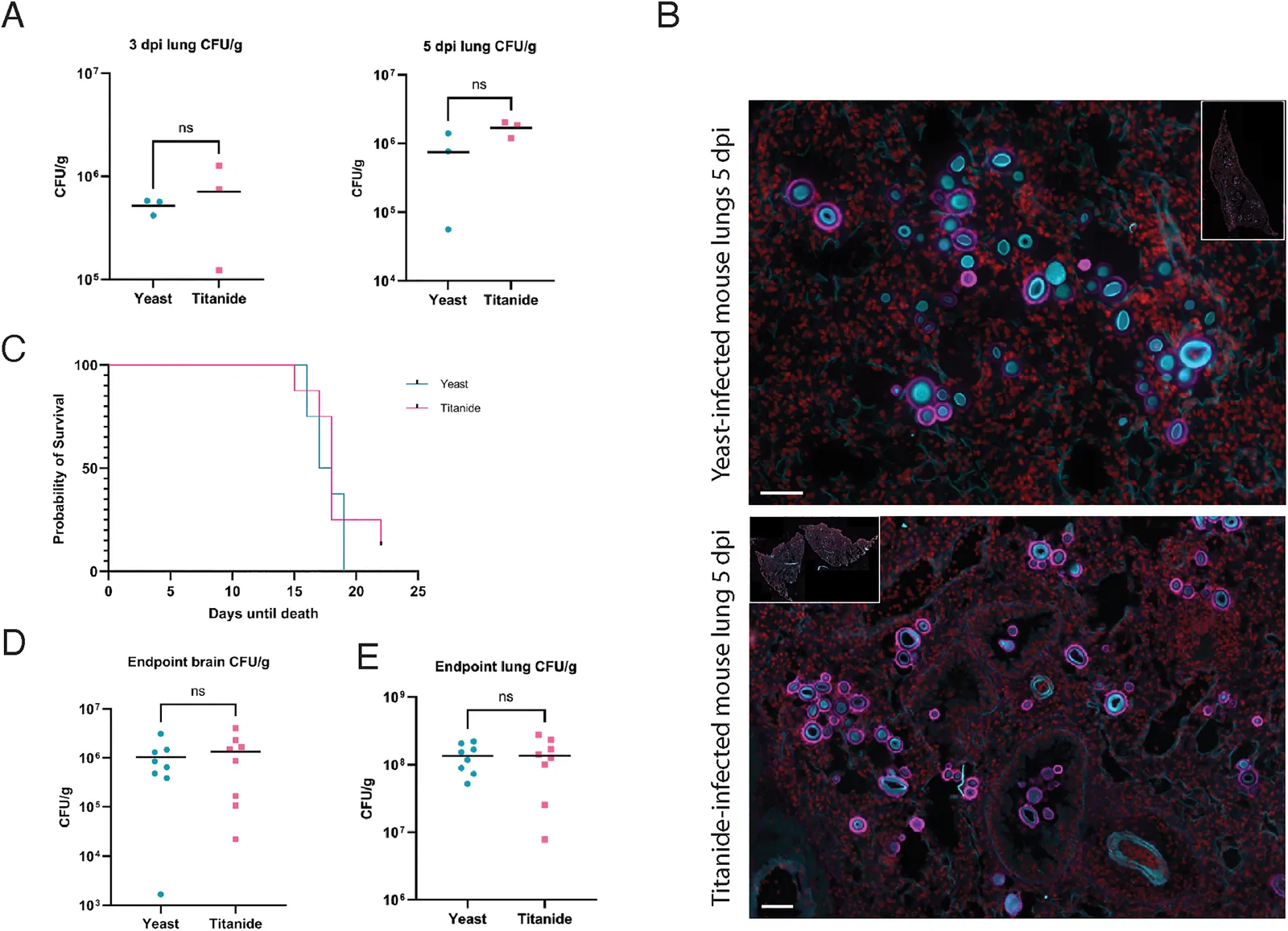
Titanides can serve as an infectious propagule and display the same pathogenic potential as yeast in a murine model. **(A)** Lung burdens (CFU/g) isolated from lungs of mice intranasally infected with an inoculum of YPD-grown yeast or titanides at 3 and 5 days post-infection (*n* = 3 mice per group). Data presented as Log CFU/g. Mean indicated by the black line. Significance assessed by Mann–Whitney *t* test. **(B)** Representative images of *C. neoformans* cell heterogeneity from histological sections taken from the mouse lung from each infection group at 5 dpi. Slices were fluorescently stained with CFW to visualise *C. neoformans* cell body and Cy5.5-mAb 18b7 to visualise capsule (PI staining to visualise all nuclei in mouse tissue). Scale bar 50 µm. Contrast adjusted for data visualisation. **(C)** Survival curve plotted over 22 days following intranasal infection of mice with either YPD-yeast or titanides (8 per group). Significance assessed by Kaplan–Meier with Mantel-Cox test. **(D)** Endpoint infectious burdens recovered from the brains of mice from **(C)**. Data presented as Log CFU/g. Mean indicated by the black line. Significance assessed by unpaired *t* test with Welch’s correction. **(E)** Endpoint infectious burdens recovered from the lungs of mice from **(C)**. Data presented as Log CFU/g. Mean indicated by the black line. Significance assessed by unpaired *t* test with Welch’s correction. The data underlying this figure can be found at https://doi.org/10.6084/m9.figshare.31171789.

Finally, to assess the pathogenic potential and virulence of titanides, we performed a longer survival assay using YPD-grown yeast and purified titanides as above (S10A Fig). Both inocula were found to be highly infectious in an immunocompetent murine model (Fig 12C). By day 22, mice in all groups had succumbed to infection, with no significant difference in the virulence between the infectious inoculum (*p* = 0.4163 as assessed by Kaplan–Meier with Mantel-Cox test) and no difference in the median survival time (17.5 versus 18 days for yeast and titanide infections, respectively) (Fig 12C). In both groups dissemination to the brain was observed, with no difference in the rate of dissemination to the brain following infection with titanides at early time points (assessed at 3 dpi, *p* = >0.9999, S10C Fig). Consistent with this, endpoint CFU recovery revealed no differences in brain or lung fungal burdens at the time of sacrifice (15–22 dpi) (*p* = 0.6154 and p = 0.9838, respectively) (Fig 12D).

Despite similar outcomes, severity scoring did reveal differences in disease progression. Specifically, mice infected with titanides displayed a delayed onset of symptoms relative to the yeast group (day 13–14 versus 10), followed by a more precipitous decline that resulted in a shortened period between start of symptoms and endpoint (3 ± 1.5 days versus 5 ± 1.7, *p* = 0.06) (S10D and S10E Fig).

To investigate differences in cytokine responses to the two infectious inoculum, lung homogenates were analysed via Luminex, a multiplex assay that allows simultaneous detection of the levels of 23 cytokines in a single sample. Cytokine profiling revealed that infection with titanides appears to induce a more eosinophilic response early in infection, notably characterised by elevated levels of eotaxin, but this difference disappears over the course of infection, with no difference in cytokine profiles observed at the time of endpoint sacrifice (Figs 13A, 13B, S11A, and S11B). Taken together, these results demonstrate that *C. neoformans* titanides are relevant for infection and can cause mortality in a murine model in a manner indistinguishable from yeast.

**Fig 13:**
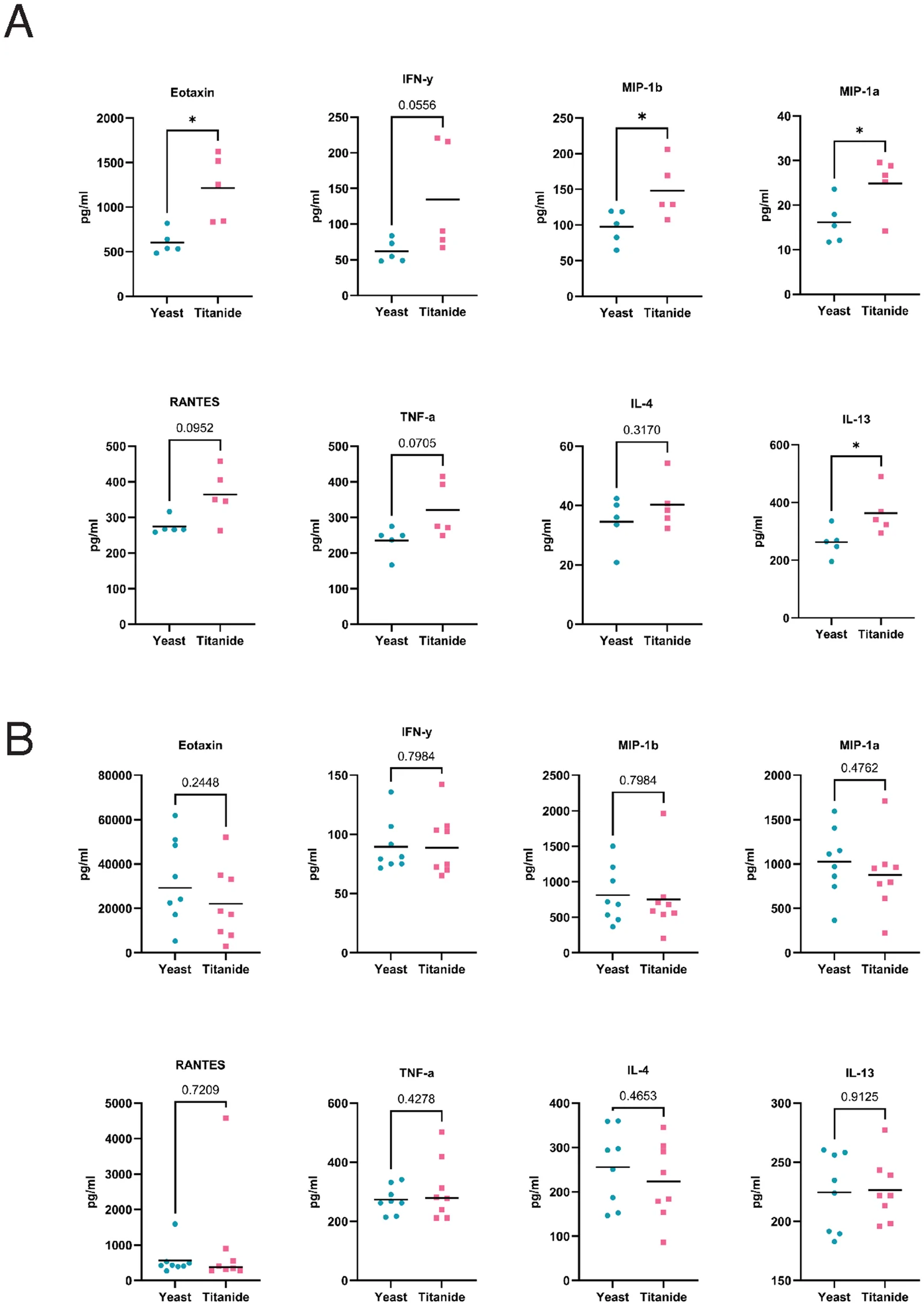
Titanides induce an eosinophilic response in a murine model early in infection. (**A**) Cytokine quantification from lung homogenate supernatant of mice intranasally infected with an inoculum of YPD yeast or titanides at 3 days post-infection. Mean indicated by black line. ‘*’, *p* = 0.0172 for eotaxin, and 0.04217 for IL-13 assessed by Welch’s *t* test. (**B**) Cytokine quantification from lung homogenate supernatant of mice intranasally infected with an inoculum of YPD yeast or titanides at the point of sacrifice. Mean indicated by black line. Normality was assessed by Shapiro-Wilk, and significance assessed by Welch’s *t* test or Mann–Whitney. The data underlying this figure can be found at https://doi.org/10.6084/m9.figshare.31171789.

## Discussion

In this work, we defined titanides, the small cell type produced by *C. neoformans* under host-relevant conditions. Beyond their size and shape, we identify major traits that define this cell type: (i) a thin cell wall and capsule with altered PAMP exposure, (ii) a lack of proliferation upon birth until stimulated to transition to yeast-like growth, and (iii) altered sensitivity to stressors including SDS, H_2_O_2_, and Amphotericin B. Importantly, they also have the ability to recapitulate infection in a mouse model, similar to infection with YPD-grown yeast.

Titanides are one of several *C. neoformans* small cell types, each with their own distinct properties [7,11,20]. Titanides form from typical cell mothers in response to host-relevant signals with a consistent timeline of emergence and are found across *C. neoformans* species complex subtypes. However, the term ‘morphotype’ includes daughter cell fate, requiring symmetric replication in that form [6]. Titanides, like titan cells, produce daughter cells that do not resemble themselves. This may indicate that titanides are a developmental cell type, as has also been proposed for titan cells [6].

Titanides are in some ways reminiscent of quiescent cells, whereby cells exit the cell cycle in response to nutrient depletion but resume growth upon a switch to rich medium [20,51–53]. However, conditions that induce quiescent cells do not induce titanides [10,54]. Like basidiospores, titanides must swell to resume growth, and have better viability after stress exposure (Fig 9C). This raises the possibility that the titanide cell type, although distinct from mating-induced progeny and asexual spores, may be a spore-like persistent form. However, spores also have distinct morphological properties [49]. Titanides lack a spore coat and have a thin cell wall surrounded by a uniform capsule, whereas spores are thick-walled with aberrant GXM-reactivity [49].

Overall, this work identifies titanides as one of many possible host-relevant fates that *Cryptococcus* typical cells can enter, along with titan and seed cells. Importantly, this suggests that typical cells are highly plastic, able to enter multiple different cell states to generate an adaptive heterogenous population. More broadly, *Cryptococcus* yeast have numerous morphology-based mechanisms of environmental stress response, including persister cells, VNBC cells, ovoid cells, and micro cells [10,21,55,56]. Morphological plasticity in response to their environment is a repeating theme for fungi, driving survival, dispersion, and pathogenesis [6,57]. Our data add to the emerging concept that *Cryptococcus neoformans* is pleiomorphic and highlight many unanswered questions. Here, we place the distinct traits of titanides within the wider morphology, environmental, and disease contexts.

### Titanides arise from a typical cell mother via asymmetric growth

We expand on the known *C. neoformans* life cycle and demonstrate that yeast can respond to titan-inducing conditions either by propagating as typical cells, transitioning into a titan cell, or generating an alternative titanide daughter. Fungi frequently exhibit morphological changes such as yeast-to-hyphal in *Candida albicans* and the thermally dimorphic pathogens [57,58]. These processes are all characterised by a direct transition of the progenitor cell into a new proliferative morphotype. In contrast, we show a distinct mode of morphogenesis wherein a typical cell gives rise to a morphologically and functionally different titanide daughter. This is the second example of asymmetric division in *Cryptococcus neoformans* typical cells: Titanides possess an altered size and shape, cell wall and capsular features, and stress and antifungal resistance properties compared to their typical cell mothers. Similarly, titan cells can also bud to produce disproportionately smaller typical-sized daughters that are morphologically distinct from their titan mothers in terms of their size, nuclear content, and antifungal resistance profiles [2,15,59]. Additionally, during mating and hyphal growth, asymmetric division in *C. neoformans* has also been observed during blastospore formation and dormant basidiospore formation from basidia [60,61]. Overall, this highlights the morphological plasticity of typical *Cryptococcus* cells and raises questions about the role of these cells in environmental and disease contexts [6].

### Environmental and nutritional context

Nutritional signals are a common trigger for *C. neoformans* morphogenesis: nitrogen starvation triggers mating and the production of sexual structures; capsule enlargement occurs in response to mannitol and nutrient starvation as well as serum, which also triggers titan cell formation [7,8,43,62–65]. High phosphate drives the transition of yeast to seed cells in vitro and in vivo [7]. Environmental hypoxia and normoxia also influence morphology: CO_2_ drives capsule production, and high CO_2_ is implicated in colony size reduction and the induction of so-called ‘ovoid’ cells, proliferative cells associated with nutrient limitation and reduced virulence [55,66].

Our data indicate that nutrient depletion alone is insufficient to trigger titanide formation. Titanides emerge after 6–7 hours and begin to dominate 48–72 hours in vitro and in vivo, which may suggest that nutrient depletion contributes to their prevalence. However, importantly, titanides do not emerge under nutrient depletion via long-term culture in rich media or during culture in minimal media [10,54]. Additionally, typical and titan cells do still proliferate under conditions that induce titanides, as demonstrated by our microfluidic imaging (S1 and S5 Movies). Instead, the molecular state of the precursor cell may be important. The switch from yeast to titan cells requires priming as well as additional environmental triggers (CO_2_, density), although the role of these signals in the generation of titanides is yet to be investigated [2,9,17]. This might include quorum sensing in these higher density cultures suppressing the formation of new titan cells in favour of titanides. Our future work will further investigate the specific signals and requirements for titanide morphogenesis.

### Titanides re-enter growth in a synchronous manner

Similar to basidiospores, titanides exhibit synchronous swelling during the switch to fresh media; Both cell types undergo a synchronous change from oval to round morphology and increase in size, resulting in a population of typical-sized cells [67]. Titanides also produce their first bud synchronously, potentially the result of reaching a size threshold for division, though synchrony is rapidly lost after the first bud. This is consistent with observations that the release of G2-arrested yeast cells into fresh rich medium triggers synchronised bud emergence for a single cell cycle, but the population rapidly becomes asynchronous [68]. Synchronous and asynchronous states can confer distinct advantages depending on the environmental conditions or context. The synchronous germination of dormant or quiescent cells likely confers a biological advantage as coordinated exit from dormancy would ensure a rapid and unified response to favourable conditions and increase the chances of survival through proliferation [69]. However, following this, a shift toward asynchronous cell cycles among progenitors and daughters may be adaptive by generating phenotypic heterogeneity within a population. This ‘bet-hedging’ strategy may be especially advantageous in fluctuating host environments, such as during immune interactions, dissemination, and antifungal exposure, or diverse environmental niches [7,70]. In this model, typical cell mothers may shift from a propensity to form titan cells after 24 hours to more frequently producing titanides at later time points, with each cell type suitable to overcome different kinds of environmental challenge. For example, titanides might resist stress through a ‘spore-like’ state while titan cells produce daughters with ploidy changes that enable adaptive growth [59]. What signals might influence this shift by the typical mother remains an open question.

### Titanide nuclear compaction is distinct from typical cells

One interesting feature of titanides is their compacted nucleus. Using a histone 4 reporter strain, we observed low signal intensity for titanides and signal consistent with G1 to G2 cell cycle progression during active proliferation. Approaches using DNA labelling dyes also reveal similar compaction, suggesting this is not simply an artefact of the reporter but reflects true structural changes in titanide genome architecture [18]. This suggests titanides undergo dynamic changes in genome compaction as G0 cells re-enter a proliferative state. Approaches such as HiC and CHiP will clarify changes in genome compaction during the return to a proliferative state.

### Titanides and disease

Upon entering the host lung, *C. neoformans* is faced with a hostile environment in which it must adapt and eventually disseminate to and survive within the CNS and brain [71,72]. Our work demonstrates that titanides can serve effectively as an infectious propagule and cause disease similarly to yeast in an immunocompetent mouse model. This is in contrast to spores and ovoid cells, which have modestly delayed virulence compared to yeast [48,55]. It is well established that during disease progression morphogenesis and heterogenous fungal responses to host-relevant signals enable a subpopulation of cryptococcal cells to enlarge, enhancing protection and persistence, while smaller cells facilitate dissemination [2,4,7,12,73,74]. Despite enrichment for small cells, we did not observe increases in dissemination to the brain at day 3, and histology on day 3 showed no difference in morphogenesis in the lungs between yeast and titanide inocula. However, we did observe alterations in markers of eosinophil recruitment on day 3, and a shift in the onset of symptoms, with titanide-infected mice having a delayed onset of symptoms and a more dramatic shift to severe disease. Eosinophil recruitment has been shown to be a determinant of disease outcome, with increased eosinophilia associated with higher fungal burdens [75,76]. The role of titanides in driving this response has not been previously reported.

In addition to altered host response, titanide stress resistance and immune evasion profiles may have implications for latency. Titanides are non-proliferative upon birth and are less preferentially phagocytosed, two features that may contribute to the establishment of a latent infection. Although it remains unclear whether titanides born in the host are non-proliferative, infection with pre-generated titanides results in a heterogenous population, which may include both new titanides and original infectious cells. Future work will address this.

With regard to therapeutic impacts, we observed similar resistance to host-relevant stress compared to YPD-grown cells. Unlike titan cells, which are more resistant to antifungals, titanides are more sensitive to Amphotericin B and nystatin, highlighting morphogenesis as key consideration for efforts to improve antifungal regimens [59]. The impact of antifungal therapy on morphogenesis may be multi-factorial, the result of both fungal stress response and the accumulation of drug-induced genetic changes. For example, clinical isolates previously exposed to antifungal therapy are associated with the subsequent formation of rare ‘irregular’ cells with aberrant morphology [22]. Our analysis of clinical isolates focussed on strains collected before antifungal exposure and found high prevalence of the capacity to form titanides across all species complex subgroups [77]. The impact of titanides on antifungal susceptibility and patient-relevant outcomes should be considered in future research.

### *Cryptococcus* is heterogeneously pleomorphic in response to environmental conditions

A striking conclusion from ongoing studies of morphology in the field has been that *Cryptococcus neoformans* is particularly pleomorphic, adapting its growth form to a wide variety of environmental conditions. This is consistent with the concept of bet-hedging previously demonstrated for *C. gattii* [78]. Titanides may offer survival advantages via several different mechanisms. First, they exhibit increased SDS resistance via paused swelling. The presence of caffeine also resulted in slowed swelling, with no impact on viability. Titanide-derived yeast appear to have an early growth advantage relative to YPD-grown yeast under ROS stress. Whether this is due to an intrinsic property of the titanide form, or via some inherited epigenetic resistance of titanide daughters remains unclear. For example, the relatively high ergosterol content and high lipid order of titanides may contribute to increased oxidative stress resistance in the first generation of daughter cells.

Uniquely, *Cryptococcus* is able to progressively induce titan cells and titanides in conditions similar to the environmental conditions it may encounter in its natural reservoirs. *C. neoformans* has been isolated from varied environmental sources such as bird guano, soil, and decaying wood niches that present the fungus with varying stressors and nutrient limitation [79–83]. The morphological changes characteristic of titanisation such as DNA replication and cell wall and capsule thickening would come at a great energy cost for the typical cell, requiring a higher nutritional environment [2]. In contrast, a non-proliferative stress-resistant titanide daughter may serve as a less energetically costly survival mechanism. Interestingly, titanides are better able to grow in YNB compared to YPD suggesting titanide morphogenesis may act as a mechanism to promote survival in limiting conditions until dissemination to an environment conducive for growth. We also observed that related non-pathogenic *Kwoniella* species can change morphology in response to 10% FCS and CO_2_ suggesting these changes are not simply a virulence determinant.

The morphology of *Cryptococcus* in the natural environment has never been reported and the exact niche of titanides is still unknown. Overall, our morphological characterisation and established definition will allow others to accurately identify titanide-related phenotypes within their work and expand our understanding of a key player in *C. neoformans* cellular heterogeneity.

## Methods and materials

### Ethics statement

Mouse infection experiments were performed under the United Kingdom Home Office project license PP9965358 and approved by the Research Ethics Committee, College of Life and Environmental Sciences, University of Exeter (eCLESBio000371 V5). We adhered to the ARRIVE guidelines for experiment design, execution, and reporting. Mice were allocated randomly to cages (4 per cage) before the groups were generated. Group allocation, infections, and monitoring for clinical sores were performed by a blinded investigator.

### Strains and media

*C. neoformans* (MATα) H99 and KN99 were a kind gift from Andrew Alspaugh [84,85]. This H99 isolate is genetically similar to H99O [31]. Cells were routinely cultured on YPD (1% yeast extract, 2% bacteriological peptone, 2% glucose, 2% bacteriological agar) plates. For routine culture, cells were incubated overnight in 10 ml YPD at 30 °C, 150 rpm. Isolate SC820 expressing fluorescently tagged histone H4 (CNAG_01648) was generated by amplifying the mNeonGreen-2× FLAG construct from plasmid pBHM2404 containing 50 bp homology regions and introducing it into KN99 using the Cas9-based method as previously described by Huang and colleagues (2022) [86]. The clr4Δ strain was a kind gift of Sandra Catania [87]. A complete list of species isolates and strains found in Fig 5 can be found in S5 Fig. Ambition clinical isolates were a kind gift from the Ambition team [77].

For titan cell and titanide induction, cells were incubated overnight at 30 °C, 150 rpm YNB without amino acids plus 2% glucose. Titan cell induction was performed in 1× PBS (–Ca^2+^, –Mg^2+^) supplemented with 10% HI-FCS at a starting OD_600_ of 0.001 at 37 °C, 5% CO_2_ as previously described [2]. To maximise the number of titanides, cultures were incubated for 72 hours, unless stated otherwise (Fig 1E and 1F). For titanide isolation, cultures were filtered through a sterile 3µm Millipore Membrane filter (Fig 1G).

Seed cells inductions were carried out according to the method outlined by Denham and colleagues (2022) [7]. Cells were incubated overnight at 30 °C, 150 rpm YNB plus 2% glucose. Seed cell induction is performed in 10% Sabouraud’s (SAB) media pH 7.4 at 37 °C, 5% CO_2_ at a starting cell count of 10^5^ cells/ml for at least 24 hours until OD_600_ ≥1.1. Cells were then sub-cultured 1:1 into fresh media supplemented with 10% potassium phosphate monobasic (7.348 mM) for 24 hours.

### Automated cell morphological and fluorescent measurements

Images were routinely acquired on an Olympus IX83 microscope equipped with 40× air objective and CoolLED pe-300 LED light source and Hamamatsu ORCA-Fusion camera. All images within a given experiment were acquired on a single day and using the same acquisition settings. For microfluidics, images were acquired using a DeltaVision Elite microscope, Lumencore 7 LED light source, and EDGE/sCMOS 4.2 camera as described below.

For automated ROI detection and fluorescent measurements where stated, images and associated metadata were analysed in FIJI, using the StarDist plugin [88]. For StarDist, Images were sum-projected to produce a 2D image from the z-stack, and 2D images segmented with a probability/score threshold of 0.5 and overlap threshold of 0.40 to detect ROIs. Images were screened before measurements were taken to remove ROIs of cut-off and non-viable cells and manually add missed ROIs. Small buds were excluded.

Where stated, fluorescence values reported were the product of Integrated Density, which accounts for differences in cell body size when measuring total fluorescence. Integrated Density is the sum of all pixel values within an ROI, calculated as the product of the region’s area and mean grey value, reflecting the total amount of signal in an ROI.

### Titanide culture population analysis

H99 cells were induced as described above in biological triplicate and imaged at 24, 48, and 72 hours. Images were analysed in ImageJ to determine the percentage of titanides based on size and shape criteria (~3 µm, oval). To obtain size, circularity, and total chitin fluorescence data for the entire population, 1 ml of 72-hour cultures were stained with 10 µg/ml Calcofluor White (CFW). Cell measurements were collected using StarDist with 3 images per biological replicate, and all cells in each image analysed for 3 biological replicates (*n* > 200 at 24 hours, >900 at 48 and 72 hours for each replicate).

For high-throughput screening of clinical and environmental isolates in S5 Fig, cells were cultured under routine titan-inducing conditions in a 96-well format. Cells were stained with 10 µg/ml CFW, imaged on an Olympus IX83 microscope equipped with 40× air objective, and cell diameter measurements collected using StarDist (See Automated cell morphological and fluorescent measurements) [88]. Scoring for the presence or absence of titanides was performed in biological triplicate, while cell diameter reports are representative of a single biological replicate with >1,000 cells per well.

### Cell surface measurements and staining

For cell wall staining, cells incubated under the indicated conditions (YPD yeast, 72-hour filtered titanides or whole titan cultures) were washed with 1× PBS and then stained with 10 µg/ml CFW, 4 µg/ml Wheat Germ Agglutinin-Alexa Fluor 594 (WGA), and 40 µg/ml Concavalin A-Alexa Fluor 488 (ConA) for 45 min at 37 °C. After staining, cells were washed twice with 1× PBS before observation by fluorescence microscopy with Z-stacks spanning 8–12 µm for filtered titanides and YPD yeast, and 14–17 µm for whole titan populations, with 1 µm slices. Chitosan levels were determined according to the protocol of Maybruck and colleagues [27]. Briefly, cells were incubated in pH 2 PBS containing Cibacron Red 4 at a concentration of 25 μg/ml at room temperature (RT) for 30 min in the dark before being washed for observation with Z-stacks spanning 5–8 µm, for filtered titanides and YPD yeast, and 14–19 µm for whole titan populations, with 1 µm slices.

For capsule staining, titan cultures were harvested after 72 hours and washed three times in PBS. Cell density was determined using a haemocytometer and adjusted to 10^7^ cells/ml, then stained with 20 μg/ml Cy5.5-conjugated mAb 18b7, or 5 µg/ml Crp127 [28]. Samples were gently mixed for 1 hour at RT, then washed three times in PBS to remove unbound antibody. For Crp127 staining, cells were further incubated for an hour with a total of 2 μg/ml Alexa 488-conjugated goat anti-mouse IgM at RT, imaged with Z-stacks spanning 12–15 µm on average with 1 µm slices [28].

For transmission electron microscopy (TEM), filtered titanide cultures, YPD-grown yeast, and seed cells were fixed and processed according to the Tokuyasu method adapted for yeast [89]. To capture the cell wall structure, an osmium tetroxide post-fixation step was included, and for ultrastructure potassium permanganate was used. Images were acquired using a JEOL JEM 1400 Transmission electron microscope.

For scanning electron microscopy, cells were fixed in 2% glutaraldehyde and 2% paraformaldehyde in 0.1 M PIPES buffer (pH 7.2) for 1 hour at RT and subsequently stored at 4 °C until further use. Cells were adhered to poly-L-lysine-coated glass coverslips for 30 min and washed three times for 5 min each in PIPES buffer. Samples were post-fixed in 1% aqueous osmium tetroxide for 1 hour, washed three times for 5 min each in deionised water, and dehydrated through a graded ethanol series (30%, 50%, 70%, 80%, 90%, and 95% ethanol for 10 min each, followed by two changes of 100% ethanol for 15 min each). Samples were then incubated in HMDS (hexamethyldisilazane; Merck, Gillingham, UK) for 10 min. Following air drying, coverslips were mounted onto aluminium stubs using adhesive carbon tabs, and the edges of the coverslips were sealed with silver paint to improve conductivity. Samples were sputter-coated with 10 nm gold/palladium using a Quorum Q159T sputter coater (Laughton, UK) prior to imaging with a Zeiss Gemini 500 scanning electron microscope.

For cell membrane ergosterol staining, whole titan cultures after 48 hours or YPD ONs were collected, and washed 1× with PBS and stained with 5ug/ml Filipin. Cells were stained for 10 min at RT in the dark and then washed 3 times with PBS before imaging. Quantification was performed on Z-stacks spanning 6–9 µm on average with 1 µm slices.

### Growth curves

Growth curve analyses were performed in 96-well format for 72-hour titanide and yeast populations. Cultures were washed with PBS and adjusted to a cell count of 2 × 10^5^ in YPD, YNB 2% glucose, or their original medium. For stress assays, 72-hour titanides and overnight YPD-grown yeast were seeded at the same density then incubated in YPD plus 0.03% sodium dodecyl sulphate (SDS), 0.5 mM hydrogen peroxide (H_2_O_2_), 5 µM caffeine, 0.5 M sodium chloride (NaCl), or 1 µM Dipropylenetriamine NONOate (DPTA NONOate). For all assays, plates were sealed with a sterile Breathe-Easy membrane and incubated in a microplate reader (Tecan Spark) at 30 °C. Growth was recorded by measuring OD_600_ at 30-min intervals for 24 hours. Following stress assays, cells were imaged using an EVOS M5000 microscope with a 40× air objective for population-level morphology, then serially diluted and plated onto YPD agar to assess viability. YPD plates were incubated at 30 °C for 48 hours then imaged.

### Microfluidics

Microfluidic devices were fabricated using standard soft lithography techniques [90]. Custom device patterns were designed in AutoCAD (Autodesk) and printed onto a chrome photomask (JD Photo Data). Master moulds with heights of 6.4 and 20.4 µm were fabricated in a clean room environment using SU-8 2005 and SU-8 2025 photoresists correspondingly. Master mould surfaces were treated with a vapour-deposited layer of trichloro(1H,1H,2H,2H-perfluorooctyl)silane (Sigma-Aldrich). Polydimethylsiloxane (PDMS) devices were cast using a 10:1 (w/w) mixture of Sylgard 184 base and curing agent (Dow Corning), degassed, and cured at 70 °C overnight. Inlet and outlet ports were made using a 1 mm biopsy punch, and microfluidic devices were irreversibly bonded to glass coverslips using a plasma machine (Zepto, Diener electronic). Flow was established using syringe pump (World Precision Instruments) and 1.01 mm PTFE tubing (Adtech, UK).

For progenitor cell experiments, cells from a KN99 ON were seeded at low density into a chamber with multiple single-cell traps each of which was made of two pillars separated by 6.75 µm distance at its narrow end and induced for 48 hours prior to imaging. Chip had a height of 20.4 µm. We imaged cells at 37 °C, CO_2_, on a DeltaVision Elite microscope at 40× dry objective or 60× oil with Z-stacks spanning 21 µm, with 3 or 1 µm Z slices. The chip was perfused at 1 µL/min with PBS 10% HI FCS plus 5 µg/ml CFW.

For titanide proliferation experiments, titanides were washed in PBS, stained with 5 µg/ml CFW, then diluted 1:10 in YPD and loaded into a custom-fabricated polydimethylsiloxane microfluidic chamber with multiple single-cell traps each of which was made of two pillars separated by 1.7 µm distance at its narrow end. Chip had a height of 6.4 µm for imaging on a prewarmed (30 °C) inverted DeltaVision Elite microscope at 40× or 60× objective with Z-stacks spanning 10 or 6 µm with 2 µm Z-slices. The chip was perfused at 1 µL/min and the cells imaged every 20 min for 5–17 hours as indicated. Typical cell control was performed using the same chip and perfusion rates, imaged every 10 min for 12 hours. Titanide swelling rate was calculated as the slope of the curve of a linear regression performed on the first 300 min of growth before cell body diameter plateaued.

### Genome content assessment

To measure the DNA content of titanides a histone H4-tagged mNeonGreen reporter strain (SC280) was induced for 72 hours, filtered for titanides, and imaged alongside YPD-grown haploid and G2-arrested cells, and YPD swollen titanides in biological triplicate. Cells were G2 arrested via O_2_ limitation as adapted from Ohkusu and colleagues (2004) [91]. Briefly, *C. neoformans* 10 ml YPD cultures were incubated in 50 ml Erlenmeyer flasks at 150 rpm, 30 °C to OD_600_ ≥ 4. Then, 30 ml YPD was added, flasks were stoppered with bungs to reduce available oxygen, and cultures incubated for a further 24 hours at 80 rpm, 30 °C before imaging at 40× dry objective with Z-stacks spanning 7–12 µm with 1 µm Z-slices. For the G2 control peak, microscope fields of view were visually inspected for an unbudded G_2_ arrest (large, unbudded) and only those ROIs measured. Fluorescence was analysed using StarDist (see Automated cell morphological and fluorescent measurements).

### Titanide daughter cell characterisation

To assess the capacity of titanides to transition into titan cells, filtered titanides were pre-stained with CFW (10 µg/ml) and inoculated at low cell density in 10% FBS in PBS for 24 hours (see Strains and media).

To identify morphological and cell wall changes following titanide transition into yeast and heterogenous culture growth, filtered titanides were incubated in their original media at 37 °C 5% CO_2_, fresh PBS 10% HI FCS at 37 °C 5% CO_2_, or YPD 30 °C 150 rpm and stained with CFW, ConA, and WGA (see Cell surface measurements and staining, and S6A Fig).

### Short term stress assay

Filtered titanides or YPD-grown yeast were adjusted to a cell count of 10^6^ and incubated in PBS supplemented with increasing concentrations of SDS, H_2_O_2_, caffeine, NaCl, or DPTA NONOate for 30 min (see Fig 9E). Cells were then washed twice in PBS, resuspended in 500µl, 5µl plated, and incubated at 30 °C for 48 hours.

### TBZ spot assay

To assess strain and morphotype sensitivity to TBZ, the indicated strains and cell types were equalised to 10^6^ cells/ml in PBS. Subsequently, 10-fold dilutions were spotted onto YPD control plates, or YPD plates supplemented with 10 μg/ml TBZ. Plates were incubated at 30 °C for 72 hours and then imaged.

### Plasma membrane lipid order measurements

Cells from 48 hrs induced titan cultures were collected and washed with 1 × PBS. Following resuspension in PBS containing 5 μM di-4-ANEPPDHQ, cells were incubated for 30 min at 37 °C. Cells were then transferred into a glass-bottomed ibidi slide. Imaging was performed on a Zeiss LSM 880 Airyscan confocal microscope equipped with a Plan-Apochromat 100× /1.4/ Oil objective at 37 °C. A 488 nm laser was selected for fluorescence excitation of di-4-ANEPPDHQ. The detection windows were set to 520–580 and 620 nm for the liquid-ordered and disordered phases, respectively. Quantification was performed using a plugin compatible with ImageJ, as outlined in reference [35]. GP values were calculated as follows: GP = (520–580 nm photon count − 620–693 nm photon count)/(520–580 nm + 620–693 nm). The experiment was performed in biological triplicate, with at least 70 cells per biological replicate, and all measurements collected on the same day to minimise the impact of variations in laser intensity on ratiometric signal analysis.

### Antifungal sensitivity testing

MIC testing methods (broth microdilution) were adapted from EUCAST guidelines. Serial dilutions of Fluconazole (128–0.24 µg/ml), Amphotericin B (16–0.02 µg/ml), and Nystatin were prepared in 2× RPMI 1640 (100 µl final volume) in 96-well plates and stored at −20 °C until use. Plates were used immediately after thawing at RT. Plates were inoculated with H99 yeast (pre-grown overnight in YPD) or titanides (isolated from 72-hour titan culture) in biological quadruplicate at a concentration of 5 × 10^4^ cells per well (200 µl final volume). Cells were resuspended alongside 100 µl of sdH_2_0 to give a final concentration of Fluconazole of 64–0.12 µg/ml, and 8–0.01 µg/ml of Amphotericin B and Nystatin. One media and no-drug growth control per replicate was included for growth inhibition endpoint determinations. Microdilution plates were incubated at 37 °C ambient air, static, for 48 hours. Microdilution plates were read using a Tecan Spark plate reader with an absorbance of 530 nm. The average values of all blanks were subtracted from readings for all wells. The MIC of amphotericin B was determined as the lowest concentration giving rise to an inhibition of growth of ≥90% of that of the drug-free control. MIC for fluconazole was defined as inhibition of growth of ≥50% of that of the drug-free control. MIC for Nystatin was defined as inhibition of 100% growth of that of the drug-free control. To assess viability following long-term exposure to antifungals, 5 µl per well of the microdilution plates were spotted onto YPD agar and incubated for 48 hours, then imaged.

### Macrophage experiments

Macrophage uptake experiments were performed using the murine macrophage-like cell line J774A.1 maintained in DMEM Glutamax supplemented with 10% heat-inactivated FBS (Gibco) at 37 °C, 5% CO_2_. Cells were used between 11th–15th passages. J774A.1 cells were seeded at 5 × 10^4^ in 200 µl in 8-well ibidi slides, and pre-incubated with 100 U/ml interferon-γ (IFN-γ) at 37 °C, 5% CO_2_ for 24 hours, then stimulated with 100 U/ml interferon-γ plus 500 ng/ml LPS for 2 hours prior to infection. Inoculum were prepared as follows: Yeast cultures were prepared by incubation in YPD at 150 rpm 30 °C and harvested after 16–18 hours. Titanides were filtered from cultures induced for 72 hours and were used within 30 min of filtering to prevent possible germination into yeast. Both YPD-grown yeast and filtered titanides were collected, washed in PBS, counted, resuspended at a density of 3 × 10^5^ cells/ml in DMEM, and opsonised for 10 min with 10 µg/ml mAb 18B7. Non-opsonised controls aliquots were prepared in the same way. Fungi were co-cultured with macrophages at a MOI of 3:1 (300 µl fungal cells at 3 × 10^5^ cells/ml per well) and imaged in live culture every 20 min at 37 °C, CO_2_, 3 images per biological replicate per time point. ‘Uptake events’ were considered to be any macrophage containing at least 1 cryptococcal cell within the first 2 hours across 3 biological replicates. Events were quantified as a percent of the total number of macrophages within the acquired images.

### Murine virulence assays

Female C57BL/6J mice (age 6–8 weeks) were purchased from Charles River Laboratories and allowed to rest for one week before commencement of experiments. The mice were housed with bedding changes every 2 days and allowed access to water and food *ad libitum* throughout the experiment. The mice were maintained on a 12 h–12 h dark–light cycle (07:00–19:00) at 20–24 °C and relative humidity of 55 ± 15% and had environmental enrichment (cardboard tube, shredded paper, and wooden blocks).

For all assays, brief inhaled anaesthesia was performed on C57BL/6 mice before intranasal inoculation with 5 × 10^5^ H99 titanide or YPD-grown yeast cells in 30 μl PBS [50]. For analysis of early dissemination dynamics, 8 mice per group were infected and sacrificed at days 0-, 3-, and 5-days post-infection (dpi) (see S10 Fig). Day 0 organs were analysed for CFUs to confirm inoculum. Tissue was homogenised in sterile PBS and then were serially diluted on YPD agar to determine fungal burdens. CFUs were determined after 48 hours at 30 °C. For all mice, the right lung was harvested for histological sectioning.

For survival assays and fungal burden analyses, 13 mice per group were infected as described above. 5 mice from each group were sacrificed 3 dpi and lung and brain examined for CFUs as described. The remaining 8 per group were monitored for morbidity and sacrificed at humane endpoint criteria, defined as signs of progressive illness (>20% gradual weight loss with unwell appearance (hunched, slow movements, piloerect, ataxic), or >20% weight loss overnight, or >30% and weight gradual loss without any other symptom, or progressive symptoms and shivering or breathing problems). Survival data was assessed by Kaplan–Meier survival curves. Left lung and brain were collected for CFUs. Right lungs were collected for histological sectioning.

### Histopathology and fungal morphology of infected organs

Harvested tissue was embedded in OCT medium, snap frozen and then stored at −80 °C. Cryostat sections (8 μm thick) were cut, transferred to slides (Epredia Superfrost Plus Adhesion), fixed with 4% paraformaldehyde, and stored at −20 °C in 90% ethanol until use. For staining, sections were washed and treated with 0.2% triton. Slides were blocked in preparation for antibody treatment with 5% (w/v) BSA V in 0.1% PBST. Tissue was then stained with Cy5.5-conjugated mAb 18b7 (5 µg/ml) suspended in 1% (w/v) BSA V in 0.1% PBST at RT for 1 hour. Finally, sections were treated with a solution of RNAseA at 10 µg/ml, propidium iodide at 1:3,000 (of a stock at 1 mg/ml), and 2 µg/ml CFW in PBS and incubated at 37 °C for 30 min in the dark, before then rinsed 3 times in PBS and imaged on an Olympus IX83 inverted microscope at 20× objective.

### Cytokine measurements

Lung homogenate was gently centrifuged at 4 °C and the supernatant collected for cytokine analysis using the Bio-Plex Pro Mouse Cytokine 23-plex Assay (Luminex-based technology; Bio-Rad Laboratories) according to the manufacturer’s instructions.

## Supporting information

S1 Fig Titanides first emerge 6–7 hours post-induction in KN99 and H99. (**A**) Representative images of KN99 and H99 following growth under titan-inducing conditions for 5, 6, and 7 hours. Scale bar 10 µm. (**B**) Percentage of titanides across 24–72 hours in KN99. All cells in a given image were measured, with 3 images per sample, in biological triplicate. Mean and standard deviation indicated.’****’, *p* = <0.0001, ‘***’, 0.0003, ‘**’, *p* = 0.0041, assessed by repeated measures ANOVA with Tukey’s multiple comparisons test. The data underlying this figure can be found at https://doi.org/10.6084/m9.figshare.31171789.
(TIF)

S2 Fig Three-micrometer filtering of 72-hour heterogenous cultures results in clean titanide population. Stitched images showing entire well of filtrate following filtering of 72-hour titan cultures as outlined in Fig 1G. A high-resolution version of this figure can be found at https://doi.org/10.6084/m9.figshare.31171789.
(TIF)

S3 Fig Contrast-adjusted titanide cells stained for key cell wall components. Representative contrasted-adjusted images of titanides stained for CFW (total chitin), ConA-AlexaFluor 488 (mannan), and WGA-AlexaFluor 594 (exposed chitin) from Fig 2C. Scale bar 5 µm. Quantification is presented in Fig 2F. The data underlying this figure can be found at https://doi.org/10.6084/m9.figshare.31171789.
(TIF)

S4 Fig Titanides display a reduced presence of the crp127 epitope. Representative images of a titanide (top), typical (middle), and titan cell (bottom) stained for cell body (CFW), and labelled for an O-acetylated moiety of GXM (Crp127 with Alexa 488-conjugated goat anti-mouse IgM secondary antibody). Scale bar 10 µm. Imaging performed in biological triplicate.
(TIF)

S5 Fig Titanide formation is prevalent across *C. neoformans* clinical and environmental isolates, but not other species. (**A**) Indicated strains were incubated under titan-inducing conditions for 72 hours in a 96-well format. Strains were assessed for presence or absence of titanides. Y indicates presence, N indicates not detected. Experiment was conducted in biological triplicate. A subset of strains were quantified, as seen in Fig 5. (**B**) Representative image of *K. pini* isolate following incubation under titan-inducing conditions for 72 hours. Scale bar 10 µm.
(TIF)

S6 Fig Titanide proliferation analysis. (**A**) Schematic outlining filtered titanide culturing conditions and staining. (**B**) Representative contrast-adjusted images of titanides following 24 hours incubation in their original media (top panel), fresh PBS 10% HI FCS (middle), or YPD (bottom) stained with CFW (total chitin), ConA-AlexaFluor 488 (mannan), and WGA-AlexaFluor 594 (exposed chitin). Scale bar 5 µm. Quantification is presented in Fig 6.
(TIF)

S7 Fig TBZ stress-testing of H4 mNeonGreen strain and different *C. neoformans* cell types. (**A**) Spot plates of SC820 strain, clr4Δ histone deacetylase mutant control, and KN99 parent on YPD 2% glucose agar containing 10 µg/ml TBZ. (**B**) Spot plates of SC820 strain YPD-grown yeast, titanides, and 24-hour titanide-derived yeast on YPD 2% glucose agar containing 10 µg/ml TBZ. The KN99 parent and clr4Δ histone deacetylase mutant were included as controls. All plates were incubated at 30 °C for 72 hours.
(TIF)

S8 Fig Representative contrast-adjusted images of histone H4 mNeonGreen SC820 strain 72-hour titanides also stained for cell body (CFW). Scale bar 10 µm. Quantification is presented in Fig 7E.
(TIF)

S9 Fig Titanides moderately better resist H_2_O_2_ stress relative to yeast. (**A**) Growth curve of 24-hour YPD yeast and 24-hour titanide-derived yeast in liquid YPD, and liquid YPD containing 0.5 mM hydrogen peroxide. Error bars represent standard deviation of three biological replicates. (**B**) Percentage growth relative to the YPD-only control of 24-hour YPD yeast and 24-hour titanide-derived yeast from (A) incubated liquid YPD containing 0.5 mM hydrogen peroxide. Error bars represent standard deviation of three biological replicates. (**C**) Comparison of endpoint OD_600_ of cells from Fig 9A and 9B in liquid YPD containing 0.5 mM hydrogen peroxide. (**D**) Comparison of endpoint OD_600_ of cells from (A) in liquid YPD containing 0.5 mM hydrogen peroxide. The data underlying this figure can be found at https://doi.org/10.6084/m9.figshare.31171789.
(TIF)

S10 Fig Titanides are an infectious propagule and have similar pathogenic potential to yeast. (**A**) Schematic of mouse work. (**B**) Representative images of *C. neoformans* cell heterogeneity from histological sections taken from the mouse lung from each infection group at 3 days post-infection, then fluorescently stained with CFW to visualise *C. neoformans* cell body and Cy5.5-mAb 18b7 to visualise capsule (PI staining to visualise all nuclei in mouse tissue). Scale bar 50 µm. (**C**) Brain burdens (CFU/g) isolated from brains of mice intranasally infected with an inoculum of YPD-grown yeast or titanides at 3 days post-infection (n = 5 mice per group). Data presented as Log CFU/g. (**D**) Clinical scores and weight loss for mice and (**E**) Comparison of time of symptom onset until death. In all panels, mean is indicated by black line. Significance assessed by Mann-Whitney. The data underlying this figure can be found at https://doi.org/10.6084/m9.figshare.31171789.
(TIF)

S11 Fig Cytokine environment in the lungs of infected mice. (**A**) Cytokine quantification from lung homogenate supernatant of mice intranasally infected with an inoculum of YPD yeast or titanides at 3 days post-infection. Significance assessed by Welch’s *t* test or Mann–Whitney. ‘*’, *p* = 0.0159. (**B**) Cytokine quantification from lung homogenate supernatant of mice intranasally infected with an inoculum of YPD yeast or titanides at point of sacrifice. ‘*’, *p* = 0.0163. Normality was assessed by Shapiro-Wilk, and significance assessed by Welch’s *t* test or Mann–Whitney. Mean indicated by black line. The data underlying this figure can be found at https://doi.org/10.6084/m9.figshare.31171789.
(TIF)

S1 Movie *C. neoformans* typical and titan cells readily proliferate during culture. 24-hour H99 cells proliferating in a microfluidics chamber in PBS 10% HI FCS at 5% CO_2_. 1 µl/min for 24 hours. Scale bar 10 µm.
(AVI)

S2 Movie Titanides swell into yeast and generate yeast progeny upon incubation in YPD for 24 hours. Concatenated movies A–C represent 3 biological replicates. 1 µl/min. Scale bar 10 µm.
(AVI)

S3 Movie Typical cells proliferate in YPD. 72-hour typical cells proliferating in a microfluidics chamber in YPD 2% glucose. 1 µl/min. Scale bar 10 µm.
(AVI)

S4 Movie Titan cell daughters are round and proliferative under the conditions from which they emerge. 48-hour H99 titan cells proliferating in a microfluidics chamber in PBS 10% HI FCS at 5% CO_2_. 1 µl/min. Scale bar 10 µm.
(MP4)

S5 Movie Titanide daughters arise from typical cell mothers after 48 hours in inducing conditions. Microfluidic-assisted live imaging of typical cells incubated in inducing conditions for 48 hours plus CFW. Arrows indicate titanide daughters. 1 µl/min. Scale bar 10 µm.
(MP4)

S6 Movie Yeast and titanide cells are not phagocytosed by macrophages in the absence of opsonin. H99 YPD-grown yeast (**A**) and filtered 72-hour titanide cells (**B**) lacking pre-opsonisation with 18b7 were cultured with J774 macrophages in DMEM plus IFN-γ for 5 hours. Minimal uptake was observed. Scale bar 10 µm.
(AVI)

S7 Movie Titanides are less preferentially phagocytosed by macrophages relative to YPD yeast following opsonisation. H99 YPD-grown yeast (**A**) and filtered 72-hour titanide cells (**B**) opsonised with 18b7 were cultured with J774 macrophages in DMEM plus IFN-γ for 2 hours. Quantification of uptake is presented in Fig 11. Scale bar 10 µm.
(AVI)

## Abbreviations

dpi: days post infection
GP: generalised polarisation
mAb: monoclonal antibodies
RT: room temperature
SDS: sodium dodecyl sulphate
TEM: transmission electron microscopy
VBNC: viable but non-culturable
WGA: Wheat Germ Agglutinin
YNB: Yeast Nitrogen Base
